# Levodopa treatment: impacts and mechanisms throughout Parkinson’s disease progression

**DOI:** 10.1007/s00702-025-02893-4

**Published:** 2025-04-11

**Authors:** Peter Riederer, Sabrina Strobel, Toshiharu Nagatsu, Hirohisa Watanabe, Xiqun Chen, Peter-Andreas Löschmann, Jeswinder Sian-Hulsmann, Wolfgang H. Jost, Thomas Müller, Johannes M. Dijkstra, Camelia-Maria Monoranu

**Affiliations:** 1 Clinic and Policlinic for Psychiatry, Psychosomatics and Psychotherapy, University Hospital Wuerzburg, University of Wuerzburg, Würzburg, Germany; 2 Department of Psychiatry, University of South Denmark, Odense, Denmark; 3 Institute of Pathology, Julius-Maximilian-University of Wuerzburg, Würzburg, Germany; 4 Center for Research Promotion and Support, Fujita Health University, Toyoake, Aichi Japan; 5 Department of Neurology, School of Medicine, Fujita Health University, Toyoake, Aichi Japan; 6 Mass. General Institute for Neurodegenerative Disease. Department of Neurology, Massachusetts General Hospital and Harvard Medical School, Boston, MA 02114 USA; 7 Am Hamburger Bahnhof 3, 10557 Berlin, Germany; 8 Department of Human Anatomy and Medical Physiology, University of Nairobi, P.O. Box 30197, Nairobi, 00100 Kenya; 9 Parkinson-Klinik Ortenau, Wolfach, Germany; 10 Department of Neurology, St. Joseph Hospital Berlin-Weissensee, Gartenstrasse 1, 13088 Berlin, Germany; 11 Center for Medical Science, Fujita Health University, Toyoake, Aichi Japan; 12 Institute of Pathology, Department of Neuropathology, Julius-Maximilian-University Ofwuerzburg, Würzburg, Germany

**Keywords:** Levodopa, Parkinson’s disease, Levodopa-induced dyskinesia (LID), OFF-phase, Long duration response (LDR), Mode of action, Progression

## Abstract

Treatment with levodopa, a precursor of dopamine (DA), to compensate for the loss of endogenous DA in Parkinson’s disease (PD), has been a success story for over 50 years. However, in late stages of PD, the progressive degeneration of dopaminergic neurons and the ongoing reduction in endogenous DA concentrations make it increasingly difficult to maintain normal-like DA function. Typically, in late PD, higher doses of levodopa are required, and the fluctuations in striatal DA concentrations—reflecting the timing pattern of levodopa administrations—become more pronounced. These DA fluctuations can include highs that induce involuntary movements (levodopa-induced dyskinesia, LID) or lows that result in insufficient suppression of PD symptoms (“OFF” phases). The enhanced fluctuations primarily arise from the loss of DA buffering capacity, resulting from the degeneration of DA neurons, and an increased reliance on levodopa-derived DA release as a “false neurotransmitter” by serotonergic neurons. In many patients, the LID and OFF-phases can be alleviated by modifying the levodopa therapy to provide a more continuous delivery or by using additional medications, such as monoamine oxidase-B (MAO-B) inhibitors, amantadine, or dopaminergic receptor agonists. Understanding the challenges faced by levodopa therapy also requires considering that the PD striatum is characterized not only by the loss of DA neurons but also by neuroplastic adaptations and PD-induced degenerations of other neural populations. This review provides a broad overview on the use of levodopa in treating PD, with a focus on the underlying science of the challenges encountered in late stages of the disease.

## Introduction

### Levodopa, a therapeutic dopamine precursor that can cross the blood–brain barrier

**Fig. 1:**
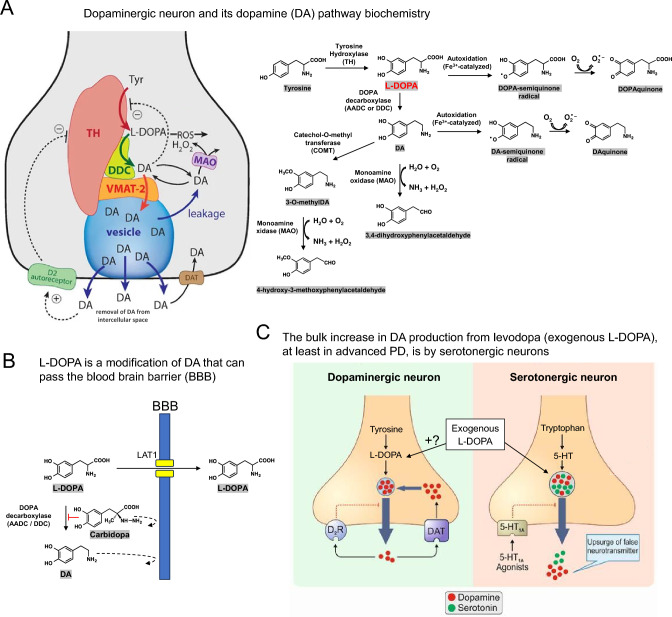
Overview of the synthesis of DA as an endogenous neurotransmitter or after conversion of exogenously administered levodopa. **A** DA is synthesized from tyrosine (Tyr) by a channelling mechanism with the enzymes tyrosine hydroxylase (TH), aromatic L-amino acid decarboxylase (abbreviated as either AADC or DDC), and the transporter VMAT2 forming a complex at the vesicular membrane. The synthesized DA is stored in synaptic vesicles for release. Significant leakage of transmitter molecules out from vesicles has been observed. Upon arrival of an action potential, vesicles are emptied, and DA is released into the intercellular space. There, DA diffuses to DA-responsive target sites or is taken up by DA transporters (DAT). TH is inhibited by DA, and there is also an inhibition of TH from extracellular DA via D2 autoreceptor signalling. Monoamine oxidase (MAO) forms hydrogen peroxide (a reactive oxygen species (ROS)) during the metabolization of DA, and also autoxidation of DA leads to the production of ROS. The formulas on the right show the biochemistry of some of the ROS and quinone-generating reactions that DA(-synthesis) can be involved in and that are believed to contribute to the sensitivity of DA neurons to PD. This figure and its legend were adapted with permission from Segura Aguilar et al. 2014, Kleppe et al. 2021, and Watanabe et al. 2024. **B** An advantage of L-DOPA over DA as a drug for treating PD is that it can pass the BBB. The transporter molecule LAT1 plays a role in this. Administered carbidopa blocks the conversion of L-DOPA to DA in the periphery. **C** In the striatum, the axons of 5HT neurons can take up exogenous L-DOPA and convert it into DA that increases the concentration of extracellular DA, at least if the number of DA neurons has diminished so that they can’t regulate those concentrations. While it has been proven that either exogenous levodopa itself or its derived DA can end up in DA neurons, there probably is no evidence showing that this leads to increased extracellular DA concentrations in the striatum, and DA neuron autoregulation mechanisms may keep a possible rise in check. This figure was adapted with permission from Farajdokht et al. (2020). Levodopa is believed to enter various cells, including neurons, involving large neutral amino acid (LNAA) transporter molecules, but the precise LNAA transporters used for entering serotonergic (5HT) or SNc DA neurons in the striatum seem not to be known (Kageyama et al. 2000; Mosharov et al. 2015)

L-3,4-dihydroxyphenylalanine is also known as L-DOPA or levodopa (levo is Latin for left) and is a biological intermediate in human dopamine (DA) synthesis from tyrosine (Fig. 1). Unlike dopamine, levodopa can efficiently cross the blood–brain barrier (BBB), and orally administered levodopa can help to correct brain DA deficits as it is locally converted by the enzyme aromatic L-amino acid decarboxylase (also known as DOPA decarboxylase; abbreviated as AADC or DDC) to DA (Riederer and Horowski 2023).

Levodopa has been the most successful drug in reducing Parkinson’s disease (PD) motor symptoms. To prevent peripheral AADC from converting levodopa into DA before it has crossed the BBB, levodopa is typically administered together with a peripheral AADC inhibitor (AADCI) that cannot cross the BBB. Common examples of these inhibitors are carbidopa (Fig. 1B) and benserazide, both of which are dopamine-like molecules. In the brain, levodopa appears to be converted to DA within dopaminergic and serotonergic neurons (Fig. 1C).

**Fig. 2:**
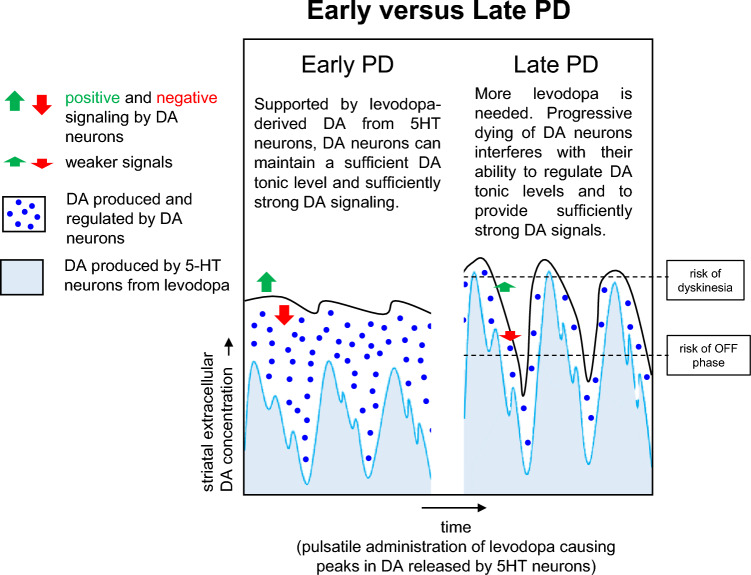
Risks of LID and OFF-phase after levodopa treatment increase in late PD. In early PD, in the striatum, if supported by some extra DA production from 5HT neurons that convert exogenous levodopa to DA as a false neurotransmitter, the remaining DA neurons are still sufficient to homogenize extracellular DA concentrations (they have a “buffering capacity”) and provide natural DA signals. In late PD, however, when the DA neurons diminish, more levodopa needs to be given so the 5HT neurons can produce more DA. Because these 5HT neurons do not reuptake DA or have any other DA-specific regulatory function, the wave of highs and lows in striatal DA concentrations starts to more exactly follow the timings of levodopa administration. These increased fluctuations increase the risk of LID and OFF-phase

PD treatment with levodopa comes with challenges, particularly as the disease progresses (Olanow and Stocchi 2018; Tran et al. 2018). These challenges include fluctuations in brain DA levels that can be too low or too high, and, if not properly managed, can, for example, lead to the return of PD symptoms between medication doses—referred to as "OFF" phases (contrasting "ON" phases)—or involuntary movements such as levodopa-induced dyskinesia (LID) (Fig. 2). It has been reported that such fluctuations occur in > 50% of PD patients within the first 5 years of levodopa treatment (Quinn et al. 1984).

Unfortunately, despite alleviating symptoms, levodopa arguably does not slow down the progression of the disease and its underlying degeneration of substantia nigra pars compacta (SNc) DA neurons. We refer to other articles for more detailed discussions on the mechanisms of PD-associated neurodegeneration. As the underlying disease cause, most models assume central roles for aggregations that include misfolded α-synuclein, such as Lewy bodies (Braak et al. 2003a, b; Riederer et al. 2023), and/or toxicity of DA-related products and oxidative stress (Riederer et al. 2019; Riederer et al. 2021; Strobel et al. 2024; Watanabe et al. 2024).

The present article focuses on the probable mechanisms underlying levodopa's effects on PD and the change therein in time. We especially discuss explanation models for the OFF-phase and LID problematics.

### History of levodopa medication

Table 1 shows an overview of important historical events in the development of levodopa as a treatment option for PD.

**Table 1:** Historical overview of the use of L-DOPA (levodopa) for treating Parkinson’s disease (PD)

**From 1913: Phytotherapy of PD**
The legumes *Mucuna pruriens* (velvet beans, “Hashou beans” in Japan) and *Vicia faba* (faba beans) were found to contain 3,4-dihydroxyphenylalanine (DOPA) in their seeds (Guggenheim 1913; Damodaran and Ramaswamy 1937). *Mucuna pruriens* is used in traditional Asian phytotherapy for treating nervous symptoms, and a recent clinical trial involving a small group of patients using Hasshou beans demonstrated relatively high efficacy and few side effects in treating PD (Sakata et al. 2024)
**From 1957: L-DOPA restores DA concentrations, which are decreased in PD**
Arvid Carlsson et al. demonstrated that DL-DOPA reversed the sedative effects of reserpine in rabbits by restoration of normal striatal DA levels (Carlsson et al. 1957). The specific distribution of DA and the decrease in the nigro-striatum in PD were found in 1959 ~ 1960 (Sano et al. 1959; 1960; Ehringer and Hornykiewicz 1960). Levodopa therapy efficiency to supplement DA deficiency in PD patients was tested by Birkmayer and Hornykiewicz (1961) in Austria and by Barbeau et al. (1961) in Canada, and a remarkable clinical efficacy by administering an adequate amount of levodopa in PD patients was already established in the late 1960s (Cotzias et al. 1967; 1969)
**From 1967: Levodopa therapy augmented with inhibitors of enzymes of DA metabolism**
The effective dose of L-DOPA could be reduced by co-administration of inhibitors of peripheral aromatic L-amino acid decarboxylase (AADC), which do not cross the blood brain barrier and thus increase the blood level of L-DOPA and the transfer into the brain (carbidopa, benzerazide) (Birkmayer and Mentasti 1967; Cotzias et al. 1969). L-DOPA therapy with monoamine oxidase B (MAO-B) inhibitors (selegiline, rasagiline, safinamide) increased the concentrations of DA formed from L-DOPA (Birkmayer et al. 1975; Reviews: Riederer and Müller 2018; Nagatsu and Nakashima 2022). Novel multitargeted iron chelators with MAO and acetylcholine esterase (AChE) inhibitory and neuroprotective properties are under clinical trial (Zheng et al. 2022). Inhibitors of catechol O-methyltransferase (COMT-I): peripherally acting entacapone, peripherally and centrally acting tolcapone, and long-acting, purely peripheral acting opicapone are employed in combination with levodopa/AADC inhibitor in PD patients (Männistö & Kaakkola 1989; Roberts et al. 1993; Review: Müller 2022)
**From 1974: Levodopa therapy augmented by DA receptor agonists**
Calne et al. discovered in 1974 the benefits of bromocriptine, a prolactin-lowering DA receptor agonist, for PD patients (Calne et al. 1974). Later, similar findings were reported for other DA receptor agonists: bromocriptine, lisuride, pergolide, cabergoline, apomorphine, piribedil, ropinirole, pramipexol, and rotigotine (Blandini and Armentero 2014). DA agonists act directly on the post-synaptic DA receptors and have a levodopa-sparing effect (Riederer et al. 2007). Combining levodopa with DA agonists delays the onset of dystonia and reduces its severity (Chen et al. 2023)
**From 1990: Levodopa delivery period extended by "controlled/ extended/delayed-release" pills**
"Controlled/extended/delayed-release" pills release levodopa more gradually after oral administration than standard “immediate release” pills (Hutton et al. 1984; Cedarbaum et al. 1990; Hauser et al. 2013). Sinemet CR, a controlled-release carbidopa/levodopa preparation, has been approved for clinical use in various countries since the early 1990s
**From 2004: Continuous levodopa supply introduced in clinical practice**
Already from the 1960s infusion methods have been tried to generate a more stable striatal DA concentration: these methods include continuous intravenous infusion (Birkmayer & Hornykiewicz 1961; Barbeau 1969; Quinn et al. 1982), subcutaneous infusion (Olanow et al. 2021; Soileau et al. 2022), and continuous intrajejunal infusion (Nyholm et al. 2003; Amjad et al. 2019). In 2004, a continuous levodopa/carbidopa intestinal gel (LCIG) infusion system was approved in Europe and introduced into clinical practice as “Duodopa”/ “Duopa”(Nyholm et al. 2013; Olanow et al. 2014)

The seminal work by Arvid Carlsson et al. (1957) demonstrated that reduced motor activity in reserpinized rabbits was due to a loss of DA that could be restored by the application of levodopa. This made it conceivable to treat PD with levodopa. Early clinical studies then demonstrated a benefit of levodopa treatment for patients with PD indeed (Barbeau et al. 1961; Birkmayer and Hornykiewicz 1961; Cotzias et al. 1967), which could be augmented by combining it with the AADCI benserazide (Birkmayer and Mentasti 1967) or carbidopa (Cotzias et al. 1969).

These findings also provided a likely explanation for why seeds of the plant *Mucuna pruriens* (velvet bean**)**, rich in levodopa, have reportedly long been used in traditional Ayurvedic medicine for treating neurodegenerative diseases like PD (Pathak-Gandhi and Vaidya 2017). Even nowadays, phytotherapy instead of pharmacotherapy for administering levodopa is preferred occasionally for a variety of reasons, supported by experimental findings (Sakata et al. 2024).

From the late 1960s, further improvements in levodopa efficacy could be realized by combining the treatment with amantadine (Schwab et al. 1969), which has antidyskinetic activity (Rascol et al. 2021), and/or inhibitors of monoamine oxidase-B (MAO-BI) (Birkmayer et al. 1975; Finberg et al. 1996; Caccia et al. 2006; Jost 2022) and/or of catechol-O-methyltransferase (COMT-I; Bonifácio et al. 2007; Rocha et al. 2013). MAO-B and COMT have a function in DA degradation (Fig. 1), and their inhibitors help to increase the levels of DA. However, these additional treatments cause their own side effects, and should be selected with care (De Bie et al. 2020; Pringsheim et al. 2021).

These various pharmacological developments served to improve and optimize levodopa therapy, and, especially, to reduce its daily dose. The latter is still important in the treatment strategy for patients with PD because levodopa can cause a variety of adverse reactions including the above-mentioned LID, but also hallucinations and other non-motor symptoms (Obeso et al. 1989; Tran et al. 2018).

The fluctuations in DA concentrations causing LID and OFF-phases in late PD (Fig. 2) are importantly caused by the bolus-type of levodopa administration (“pulsatile” administration) in combination with the short half-lives of levodopa and DA. Therefore, already since the 1960s other methods have been tried to provide a more stable striatal DA concentration. These methods include oral preparations that release the levodopa more gradually after oral administration than the standard “immediate-release” pills and alternatively are called "controlled-,” “extended-,” or “delayed-release" pills. Compared to the standard medication, they reduce the fluctuation in striatal DA concentration, but have the disadvantage of a slower relief from PD symptoms (Hutton et al. 1984; Cedarbaum et al. 1990; Hauser et al. 2013; Poewe and Antonini 2015). Extended-release preparations have been introduced into clinical practice from around 1990.

Also, methods have been reported that apply continuous intravenous infusion (Birkmayer and Hornykiewicz 1961; Barbeau 1969), subcutaneous infusion (Olanow et al. 2021; Soileau et al. 2022), or continuous intrajejunal infusion (Nyholm et al. 2003). The latter has become commercialized as “Duodopa” (or “Duopa”) in 2004 in Europe, and later elsewhere, and is successfully used by a small percentage of PD patients who wear a portable pump.

More than 50 years after its introduction, and despite the complications after prolonged use, levodopa-based therapies are still regarded as the gold standard of PD treatment (Lees et al. 2023).

### Why levodopa can work: dopamine in the striatum is tonically released by volume transmission

Signalling by some neurotransmitters, such as glutamate or gamma-aminobutyric acid (GABA), is mostly restricted to precise neural connections at synapses. That type of signalling can be called “wiring transmission.” However, DA is mostly released by extrasynaptic “volume transmission,” in which DA diffuses from the release site to multiple target sites (Arbuthnott and Wickens 2007; Fuxe et al. 2013). This volume transmission character is further enhanced by the SNc DA neurons—of which the total number is relatively small with only several hundred thousand in a human brain—being able to “wire and fire together” (Vandecasteele et al. 2005; Joshua et al. 2009; Eshel et al. 2016) and having huge axonal arbors in the striatum (Matsuda et al. 2009) where they release DA not only from synapses but also from varicose “bead” structures (Liu et al. 2018; 2021). Moreover, this volume release is tonic (continuous), supported by a robust “pacemaker” system (Tubert et al. 2023), and DA signalling in the striatum is essentially based on decreases or increases of DA concentrations compared to the tonic level (Fig. 2) (Eshel et al. 2016; Wang et al. 2021). Generally, the DA release in the striatum provides a signal that stimulates activities and processes, of which, for the dorsal striatum, motor activities are the best known (Wise and McDevitt 2018; McCutcheon et al. 2019). More or less, DA signalling in the striatum through SNc DA neurons provides a green or red traffic light for actions or processes to proceed, predominantly through overall striatal DA concentrations and to a far lesser extent through the precise delivery of DA to selected circuits of neurons. For this reason, the bulk increase in tissue DA levels in PD patients by levodopa medication is helpful, as it helps to increase DA concentrations to physiological background levels against which other (DA) signalling can operate.

### Sustained treatment with levodopa is not necessarily problematic, as exemplified by Segawa disease

Segawa disease, also known as dopa-responsive dystonia, offers evidence that levodopa treatment for overcoming DA deficiencies does not necessarily lead to the treatment-associated difficulties observed in the later stages of PD. First discovered by Masaya Segawa (Segawa 1971; 2011), this disease—typically presenting with dystonia and gait disturbances from childhood—is characterized by genetic deficiencies that significantly reduce DA production. Most commonly the genetic defect is in the *GTP cyclohydrolase I* gene (*GCHI* deficiency; Ichinose et al. 1994), the product of which supports tyrosine hydroxylase (TH) function (for TH function see Fig. 1), but the deficiency can also be attributed to other genes such as the *TH* gene itself (*TH* deficiency; Lüdecke et al. 1995). Decreases in putaminal DA in the range from 44% (asymptomatic) to 88% (symptomatic) have been reported (Furukawa et al. 1999; Furukawa et al. 2002), which overlaps with the levels of reduction seen in PD (see below). However, a critical difference with PD is that Segawa disease is not associated with progressive neurodegeneration. This likely explains why lifelong treatment with low levels of levodopa (e.g., about 20 mg/kg/day of plain levodopa or 4–5 mg/kg/day with AADC inhibitor) sustainably and dramatically alleviates motor symptoms. The absence of serious side effects appears to be common among levodopa-treated Segawa disease patients (Segawa 2011).

## The problem levodopa has to overcome: the progressively increasing DA deficit in PD

### A core characteristic of PD is the progressive and preferential demise of SNc DA neurons and the resulting decrease in striatal DA concentrations

PD is a devastating neurological disorder that as its “core characteristic” shows progressive preferential degeneration of DA neurons in the SNc, although other neurons are also affected (Braak et al. 2003a, b; Jellinger 2019; Watanabe et al. 2024). SNc DA neurons release their DA predominantly through projections to the dorsal striatum (“striatum” from here), but also to other regions including the nucleus accumbens (part of the ventral striatum), which may help explain some of the non-motor disease phenotypes associated with PD (Björklund and Dunnett 2007; Mavridis et al. 2011; Hanganu et al. 2014). Furthermore, through somatodendritic DA release, the SNc DA neurons also affect the SN pars reticulata (SNr), and reduction in this SNc-to-SNr signalling has been postulated to play an important role in PD as well (Surmeier et al. 2023). However, the best-known critical symptom in PD concerns the decrease of striatal DA concentration below a threshold necessary for normal engagement in various motor activities, leading to characteristic symptoms such as tremor, rigidity, bradykinesia, and postural instability (Moustafa et al. 2016). In this review, we will mostly focus on the changes in the DA concentration in the striatum, because it is the best quantified and understood region of DA release by SNc DA neurons.

### Percentages of SNc DA neuron losses and reductions in striatal DA concentrations during PD

Idiopathic PD is a disease of the elderly, who even without PD show a gradual loss of SNc DA neurons, and PD can —to an extent— be considered as accelerated aging (Scherman et al. 1989; Fearnley and Lees 1991; Hörtnagl et al. 2020; Watanabe et al. 2024). The DA system in healthy young people appears to have a surplus of DA for the proper performance of motor functions at a gross level, such that the typical PD symptoms emerge only after very considerable decreases in SNc DA neurons and striatal DA concentrations.

Neuropathological research and imaging studies have been performed to assess the loss of SNc DA neurons and the decrease of DA concentrations in striatum, putamen, and caudate nucleus. Respective data demonstrate about a 60–80% loss of dopaminergic neurons in the SN and a loss of striatal DA by 60–98% at the time of advanced/final stages of PD (Bernheimer et al. 1973; Kish et al. 1988; 2008; Scherman et al. 1989; Fearnley and Lees 1991; Pakkenberg et al. 1991; Pifl et al. 2014) (Table 2). However, the loss of DA neurons in PD at the time of symptom onset is at a range between 30 and 70% only (Fearnley and Lees 1991; Ma et al. 1997; Marsden 1990; Lang and Lozano 1998; Dauer and Przedborsky 2003; Ross et al. 2004; Greffard et al. 2006) (Table 2).

**Table 2:** Loss of SNc DA neurons and striatal DA in PD

1. Loss of DA neurons in PD at symptom onset (as summarized by Cheng et al. 2010)		
Loss of pigmented neurons in SN	31%	Fearnley and Lees (1991)
Loss of pigmented DA neurons in SN	30%	Ma et al (1997)
Loss of neurons in SNc	30%	Greffard et al. (2006)
Loss of SNc neurons	70%	Lang and Lozano (1998), Dauer and Przedborski (2003)
Loss of SNc neurons	50%	Marsden (1990), Ross et al. (2004)
2. Loss of striatal DA neuron terminal markers at symptom onset (as summarized by Cheng et al. 2010)		
[^3^H]-TBZOH binding ofvesicular monoamine transporter in the CN	49%	Scherman et al. (1989)
DAT-binding in striatum using ß-CIT	39 – 51%	Tissingh et al. (1998)
PET [^11^C]-MP Putamen	56 – 71%	Lee et al. (2000)
SPECT [^123^I]-IPT	43%	Schwartz et al. 2004
VMAT2 PET [^11^C]-DTBZ	51—62%	Lee et al. 2000
Loss of putaminal [^18^F]-DOPA uptake	X = 33%	Morrish et al. (1995);(1998) Lee et al. 2000 Hilker et al. (2005)
3. Loss of DA neurons in advanced stages of PD		
Loss of DA neurons in SN	60–80%	Fearnley and Lees (1991) Pakkenberg et al. (1991) Damier et al. (1999) Rudow et al. (2008)
Loss of putaminal DA 1988; 2008	60–98%	Bernheimer et al (1973) Kish et al. (1988; 2008) Pifl et al. (2014) Scherman et al. (1989)

*SN* substantia nigra; *SNc* SN pars compacta; *DA* dopamine; *TBZOH* tetrabenazine (TBZ) or its hydroxylated metabolites; *CN* caudate nucleus; *DAT* dopamine transporter; *β-CIT* [^123^I]β-CIT (2β-carbomethoxy-3β-(4-iodophenyl)tropane); *PET* positron emission tomography; MP, methylphenidate; *SPECT* single photon emission computed tomography; *IPT* N-ω-fluoropropyl-2β-carbomethoxy-3β-(4-iodophenyl)tropane; *VMAT2* vesicular monoamine transporter 2; *DTBZ* dihydrotetrabenazine

Decreases in DA concentration in the caudate nucleus of two patient cohorts with different ages at PD onset were 68% and 82% (Riederer and Wuketich 1976). Imaging studies of the caudate nucleus measuring vesicular monoamine transporter 2 (VMAT2) binding of tritiated α-dihydro tetrabenazine showed that motor symptoms tend to be recognized in clinical practice only after an approximately 49% decrease in binding sites (data from Scherman et al. 1989 analysed by Cheng et al. 2010). PD imaging studies, as summarized elegantly by Cheng et al. (2010) for studies on dopamine transporter binding using positron emission tomography (PET) and single photon emission computed tomography (SPECT) technology, show a loss of striatal binding by 59% (mean of three studies by Tissingh et al. 1998; Lee et al. 2000; and Schwartz et al. 2004). PET-studies by Lee et al. (2000) on VMAT2 binding showed a decrease of around 56.5% (mean value) and thus agreed with the findings of Scherman et al. (1989). However, the percentage of loss in putaminal ^18^F-DOPA by about 33% (mean of four PET-studies: Morrish et al. 1995; 1998; Lee et al. 2000; and Hilker et al. 2005) is less dramatic at the time of symptom onset.

Discrepancies between studies may depend on the age of onset of PD as shown by Riederer and Wuketich (1976), and also on the use of different technologies, problems for quantification, PD subtypes with variation of disease progression, the patient's sensitivity to the onset of symptoms, subareas of interest, drug treatment before imaging studies, duration of coma, limited binding specificities of ^18^F-DOPA (e.g., binding to AADC located in dopaminergic neurons, glia, and in serotonergic axonal fibres terminating in striatal areas), etc. However, although the precise percentages and their relevance can be debated, it is clear that typical PD symptoms only occur after major losses/reductions in SNc DA neurons and striatal DA concentrations, and that these deficits increase as PD progresses.

### The regional progression of SNc DA neuronal degeneration

The somatotopic pattern of dopaminergic terminal loss is most severe in the dorsal part of the putamen at the early stage of PD but extends to the ventral putamen and caudate nucleus at later stages (Jellinger 1991; 2019).

Neuronal dysfunction of the SN as measured by tyrosine hydroxylase (Mogi et al. 1988) and dopamine transporter (DAT) immunoreactivity precedes degeneration (Jellinger 2019). Surmeier et al. (2017) described a 25% loss of DA neurons in the dorsal tier of the SN, while observing a near-total loss in the ventrolateral part.

At both PD motor symptom onset and death, the loss of striatal DA markers exceeds that of SNc DA neurons (Cheng et al. 2010). This has been used for proposing a “dying-back process” (retrograde degeneration) in PD of SNc DA neurons (Bernheimer et al. 1973; Hornykiewicz 1998), as has been—debatably because protein synthesis is mostly controlled by the cell body—the early decline in axonal transport motor proteins (Chu et al. 2012). Because clear pathogenic observations in SNc DA neurons are first observed in their axonal arbour in the striatum and only later in the SNc itself, PD is sometimes named a “synaptopathy” (Longhena et al. 2017; Imbriani et al. 2018; Bridi and Hirth 2018). However, it has also been argued that the root causes of the degeneration of SNc DA neurons—of both their axons and cell bodies—more likely map to their cell bodies in the SNc because of local stresses and the more likely intracellular direction of impact (Watanabe et al. 2024).

### Compensatory mechanisms in the striatum in the early phase of PD

Many researchers believe that compensatory factors during presymptomatic and prodromal PD can explain why typical motor symptoms do not manifest until after very serious losses of DA neurons and striatal DA (e.g., Bernheimer et al. 1973; Sossi et al. 2004; Cenci 2014; Jastrzębowska et al. 2019). For example, Blesa et al. 2017 stated in their frequently cited review on this matter: “*The cardinal features of PD become clinically obvious only after an extensive population of dopaminergic neurons is lost, usually around 50–60% and striatal DA concentration falls below 70% approximately. Thus, compensatory mechanisms must be* [underlining by us] *operating in the early phase (pre-symptomatic) of PD to allow such marked depletion to take place without symptomatic manifestations.*” However, in that same paper, Blesa and coworkers also stated: “*Despite all of the above, the pathophysiology of compensatory mechanisms in PD remains unclear, and indeed, has not been fully explored.*” Counterarguments to the supposed need for an explanation through compensatory mechanisms are: (i) in most if not all animals the striatal DA concentrations may be lower than in humans (Raghanti et al. 2018; Watanabe et al. 2024); (ii) high striatal DA concentrations might only be beneficial for non-essential functions like, for example, cognitive or emotional learning (Mozley et al. 2001; Badgaiyan 2010); and (iii) sensitive motor movement analysis can detect changes caused by prodromal PD up to seven years before clinical diagnosis of PD (Schalkamp et al. 2024).

Nevertheless, the brain is very plastic, and some compensatory mechanisms presumably exist in the striatum during prodromal—and also later phases of—PD.

#### Compensatory changes in DA metabolism and turnover by SNc DA neurons may or may not exist

Zigmond et al. (1990) concluded that studies using a rat 6-hydroxydopamine (6-OHDA) model of PD suggested increased synthesis and release of dopamine (DA) from remaining SNc DA neurons, together with a reduced rate of DA inactivation. However, if such compensatory mechanisms would exist in human PD—for which there is no convincing evidence—they cannot be efficient because the strong reduction in striatal DA is characteristic of PD, even in the prodromal phase. Furthermore, such increased DA synthesis and release would be hard to reconcile with the fact that DA production by individual SNc DA neurons in a healthy brain is already considered very high (even said to be “near a bioenergetics cliff”) and a major reason for the cell’s vulnerability to PD (Surmeier et al. 2012; Watanabe et al. 2024).

Some studies may have been overinterpreted by authors—or later other scientists—as representing (suggestive) evidence for compensatory mechanisms. For example, Sossi et al. (2004) found by PET scan that the turnover rate of injected ^18^F-fluorodopa was considerably lower in PD patients than in healthy controls. Nevertheless, they considered PD-stage-dependent differences in the ratios of these turnover rates between putamen and caudate to suggest the existence of compensatory mechanisms. However, such ratio differences might also be explained by regional differences in the patterns of neurodegeneration progression.

Another proposed striatal compensatory mechanism has been the reduced uptake of extracellular DA by SNc DA neurons through downregulating (the activity of) the DA transporter (DAT) molecules of these cells, but typical DAT monitoring data only analyse DAT expression per tissue so that they cannot distinguish whether individual SNc DA neurons downregulated DAT for compensation (e.g., Wile et al. 2017).

Summarizing this topic in their review, Blesa et al. (2017) concluded, and we see no reason to disagree: “*There may be changes in striatal DA metabolism and turnover in the presymptomatic stage of PD, but these changes are most likely subtle and difficult to detect using standard biochemical approaches or traditional assays.*”

#### Compensatory changes in striatal medium spiny neurons (MSNs)

In PD, SNc DA neurons are under stresses that lead to their progressive degeneration, so it might be more logical if in the striatum other cell types—that are less stressed—provide adaptive changes to compensate for the gradual decrease in DA concentrations. Indeed, it seems easier to observe compensatory changes in other neurons than in SNc DA neurons themselves, especially in MSNs.

**Fig. 3:**
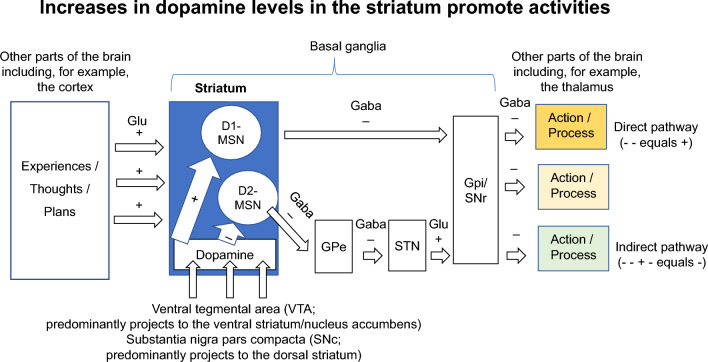
Increases in DA levels in the striatum promote activities by stimulating circuits that include D1 MSNs and inhibiting circuits that include D2 MSNs. Glutamatergic (stimulatory) neurons from different brain regions and from many different neurocircuits innervate the striatum. Here, neural activities are validated by DA that is released by dopaminergic neurons innervating from the ventral tegmental area (VTA) or the substantia nigra (SN). In the striatum, DA stimulates medium spiny neurons (MSNs) that express dopamine receptor D1, so-called D1-MSNs, and inhibits D2-MSNs. The activated D1-MSNs, which are GABAergic (inhibitory), relay through the substantia nigra pars reticulata (SNr) or globus pallidus externa (GPe) to inhibit the inhibition of neural activities that here are described as “Action/Process”; this stimulatory pathway is called the “direct pathway.” If striatal DA levels decrease, this stimulates D2-MSNs, which are also GABAergic and relay through the globus pallidus externa (GPe), subthalamic nuclei (STM), and GPi/SNr to form the “indirect pathway” that inhibits (because “ − − + − ” equals “ − ”) neural activities (“Action/Process”) that can be stimulated by the direct pathway. This figure and its legend were adapted with permission from Dijkstra and Nagatsu 2022

DA signals in the striatum are importantly received by MSNs, which to this end, generally (although this is a simplification), either express D1 receptors that convert DA binding into cell activation, or D2 receptors that convert DA binding into cell inactivation. The D1 MSNs project directly to the globus pallidus internus (GPi) and SNr, and participate in the “direct pathway” that generally promotes various actions/processes, whereas the D2 MSNs form part of the “indirect pathway” that first projects to the globus pallidus externus (GPe) and the subthalamic nucleus (STN) before delivering opposing signals to those of the direct pathway in the GPi/SNr (Fig. 3) (Surmeier et al. 2007). A lack of sufficient DA concentration reduces the promotion of various activities/processes through the direct pathway while increasing the inhibition of activities/processes through the indirect pathway. In PD, most severely affected are the SNc DA neurons that predominantly (but not only) project to the dorsal striatum and there play a major role in promoting motor activities. However, similar DA-mediated circuitries are found in the nucleus accumbens, which receives DA predominantly from the VTA (Fig. 3) and is especially important for mental processes such as validation and motivation.

For decades it has been known that DA-deprived D1 MSNs and D2 MSNs become hypersensitive to D1-receptor and D2-receptor agonists, respectively, including levodopa-derived DA (Chase et al. 1973; Trugman and James 1992). For example, in DA deficient mice, the D1-receptor agonist SKF 81297 and the D2-receptor agonist quinpirole caused three-to-six-fold and five-to-six-fold increases, respectively, in locomotor activity compared to the responses elicited in wild-type (wt) mice, and treatment with levodopa even caused a 13-fold increase in locomotor activity (Kim et al. 2000). The steady-state DA receptor densities appeared to be unaltered in this study, and other mechanistic explanations are required (Kim et al. 2000).

However, it has also been reported that D1- and D2-receptor densities are augmented as a compensatory response to the striatal dopamine deficit in PD (Hassan and Thakar 1988), while levodopa therapy was found to downregulate the elevated D2 receptor densities (Guttman and Seeman 1985). This would provide a straightforward mechanical explanation for the adaptation of MSN DA-sensitivity to the concentrations of DA available.

In PD, dystrophic changes have been reported in the dendrites of MSNs, with a loss of dendritic length and dendritic spine number (McNeill et al. 1988; Zaja-Milatovic et al. 2005; reviewed by Deutch et al. 2007). At a closer look, in a mouse PD model, only the D2 MSNs showed a loss of dendritic spines and glutamatergic synapses in response to DA depletion (Day et al. 2006). These changes in D2 MSNs would make biological sense, because their lessened sensitivity to stimulation by glutamate could help avoid their overactivation under low concentrations of (their inhibitor) DA.

In disagreement with the above, some other studies suggest that in PD not only D2 MSNs but also D1 MSNs show dendritic atrophy, while the densities of D1 and D2 receptors increase (reviewed by Blesa et al. 2017).

A recent article claimed that the data presented on MSN dendritic atrophy in human PD patients—in the five studies they analysed—have been limited and conflicting (Zhong et al. 2023). The same study also found that DA depletion does not trigger MSN dendritic atrophy in mice, and therefore speculated that—if true—the reported MSN dendritic atrophy in PD may be a component of neurodegeneration in PD rather than a consequence of DA denervation (Zhong et al. 2023). Nevertheless, the study by Zhong et al. (2023) did agree with DA depletion leading to hypersensitivity of DA receptors.

In short, D1 MSN and D2 MSN appear to adapt to low DA concentrations by becoming more sensitive to DA, which is consistent with the general fact that their plasticity—a process that enables learning and the formation of habits—is controlled by the level of their stimulation (Surmeier et al. 2007; Lovinger 2010). However, there is conflicting information and ongoing debate about the underlying mechanisms of these adaptations in PD.

#### Striatal serotonergic neurons: degeneration but probably also some compensatory mechanism

Serotonergic (5HT) neurons, the cell bodies of which reside in the raphe nuclei, project to various brain regions, including the striatum, to release 5HT (serotonin) (Hensler 2006). The raphe nuclei are one of the brain regions where Lewy bodies and cell loss are often found in the early stages of PD (Jellinger 1991; Braak et al. 2003a, b). A gradual decrease—though far less dramatic than in dopaminergic markers—in serotonergic markers in the striatum has been implicated in PD progression, affecting both motor and non-motor functions that depend on serotonin (Politis and Niccolini 2015; Pagano et al. 2017a, b). However, beyond this gradual decline, there are indications of compensatory mechanisms involving 5HT neurons. Namely, in Leucin-Rich Repeat Kinase 2 (*LRRK2)* gene mutated PD, increased serotonin transporter binding in the striatum, brainstem, and hypothalamus during the prodromal phase has been observed and was suggested as a compensatory mechanism (Wile et al. 2017).

#### Other proposed compensatory mechanisms

Various other non-dopaminergic and cerebral mechanisms have been suggested to adapt to the progressive striatal dopamine depletion in PD. These include:

*Shifts in tyrosine hydroxylase (TH) expression.* A transgenic parkinsonian mouse model reported a two-fold increase in striatal neurons expressing the *TH* gene (Troshev et al. 2023). Such mechanism, coupled with an elevation of TH enzymes in the neuronal cell bodies and nerve fibers, has been postulated to counteract striatal DA depletion in PD (Troshev et al. 2022). However, whether striatal TH^+^ neurons make a contribution to DA production is debated (Xenias et al. 2015).

In the PD striatum also increases were observed in the proportions of both the TH isoform 2 and activated TH (Ser40-phosphorylated), and the latter has been proposed as a compensatory mechanism to increase DA production (Shehadeh et al. 2019). However, it was not clarified whether these shifts might involve a higher survival of striatal TH^+^ neurons compared to SNc DA neurons.

*Enhanced cholinergic signalling.* PD is associated with dysregulation of cholinergic signalling, and both hypocholinergic and hypercholinergic activities have been observed (Bohnen et al. 2022). Enhanced cholinergic neuronal activity in the mesopontine tegmentum in PD may represent a rescuing effort in the early prodromal stages of the illness. On the other hand, some motor deficits/abnormalities have been related to acetylcholine depletion in various areas of the brain, including the caudate nucleus, thalamus, and cerebellum. This offers another promising pharmacological approach to managing PD's gait and postural difficulties.

*Cortical plasticity and motor adaptation.* During action selection, PD can impede motor recycling (Fritsche et al. 2020). Thus, switching motor plans to a new action may challenge these patients. However, it has been suggested that action selection is maintained to some degree by compensatory cerebral mechanisms (Redgrave et al. 2010). This cerebral compensation can be achieved through adjustment processes, such as selective recruitment of processes designated for other actions or upregulation of mechanisms dedicated to that task (Cabeza et al. 2018). These are proposed to be operative during the pre-symptomatic phase of the disorder (Helmich et al. 2015).

In addition, the deficient input from subcortical areas is compensated by augmenting cortical facilitation and decreasing cortical inhibition (Rothwell and Edwards 2013). This demonstrates cortical plasticity in PD. Also, the unaffected /less affected putamen exerts a compensatory role by reinforcing the cortical output, thereby improving motor control (Blesa et al. 2017).

However, as the disease progresses, the protective compensatory mechanisms succumb to the relentless progression of the neurodegeneration. Indeed, the prolonged dysfunction of dopamine release contributes to the manifestation of the clinical features of the disease. This is probably due to the inability of the compensation mechanisms to overcome the progressive cellular dysfunction (Cramb et al. 2023).

Interestingly, levodopa treatment may weaken the compensatory hyper-connectivity in the brain (Huang et al. 2019).

## Current practices for levodopa therapy in PD

### Levodopa therapy guidelines and practices

Table 3 summarizes current guidelines and practices for levodopa treatment in PD. One of the important sources is the guidelines by the American Academy of Neurology (AAN; Pringsheim et al. 2021). Clinicians should counsel patients with early PD on the benefits and risks of therapy with levodopa, DA (receptor) agonists, or MAO-B inhibitors (Pringsheim et al. 2021). Levodopa treatment gives the greatest benefit for motor symptoms but has a risk of dyskinesia at higher doses (Katzenschlager et al. 2008; PD Med Collaborative Group et al. 2014; Pringsheim et al. 2021). Levodopa is usually co-administered with an AADC inhibitor in tablet form. High doses of levodopa (> 600 mg/day) carry a considerable risk of LID (Olanow and Stocchi 2018), and levodopa treatment of patients with early PD should be titrated from low (usually around 150–300 mg/day) to higher doses to find the lowest effective dose (Pringsheim et al. 2021).

**Table 3:** Summary of guidelines for levodopa treatment of PD

1. Indications for Levodopa:
Generally, in patients with early PD who seek treatment for motor symptoms, levodopa is the initial preferential dopaminergic therapy
2. Initiation of Therapy, Dosage, and Administration:
The combination of levodopa with a decarboxylase inhibitor is standard practice (one of the common combinations is 100 mg levodopa is with 25 mg DDI)
Levodopa should be started at a low dose and titrated slowly to minimize side effects. Initial dosing typically starts with half or complete tablets of 25mg/100mg (carbidopa or benserazide)/levodopa, taken two to three times daily
Levodopa dosage should be adjusted based on patient response, with effective daily doses usually ranging from 300 to 800 mg, divided into multiple doses
With PD progression, higher levodopa doses tend to be needed. In patients with severe recurrent OFF periods, daily requirement could be around 2000 mg
Clinical trials in early PD demonstrate symptomatic benefit with levodopa/DDI at dosages of 150–300 mg/day and a lower risk of dyskinesia with dosages < 400 mg/day
2. Managing Side Effects:
Common side effects include nausea, dizziness, and orthostatic hypotension. These can often be managed by adjusting the dosage or timing of administration
Use antiemetics like domperidone to manage nausea if necessary
Monitor for and manage dyskinesias (involuntary movements) by adjusting the dose or adding medications like amantadine
3. Motor Fluctuations:
Patients may develop motor fluctuations such as "wearing-off" (end-of-dose effect) or "ON–OFF" phenomena with long-term use
Adjust the timing and dosage of levodopa or consider using extended-release formulations
Additional medications (e.g., DAs, MAO-B inhibitors, COMT inhibitors) can help provide more stable dopaminergic stimulation
4. Advanced PD Management:
Consider continuous dopaminergic stimulation methods (e.g., intestinal gel infusion) or deep brain stimulation for patients with advanced PD and refractory motor complications
5. Non-Motor Symptoms:
Levodopa can improve some non-motor symptoms of PD, such as mood and sleep disturbances. Specific treatments for non-motor symptoms should be considered as needed

This table summarizes information from: “Levodopa in the treatment of Parkinson's disease: current status and new developments” by Salat and Tolosa 2013; “Parkinson Disease” by Halli-Tierney et al. (2020); “A Report of the AAN Guideline Subcommittee” by Pringsheim et al. (2021); the Mayo Clinic (https://www.mayoclinic.org/drugs-supplements/carbidopa-and-levodopa-oral-route/proper-use/drg-20095211); and “Initial pharmacologic treatment of Parkinson disease” by Spindler, 2024, at UpToDate (https://www.uptodate.com/contents/initial-pharmacologic-treatment-of-parkinson-disease)]. DDI, aromatic amino-acid decarboxylase inhibitor

### Levodopa therapy during the early PD stage

The Earlier vs Later Levodopa Therapy in Parkinson Disease (ELLDOPA) showed that during the first 40 weeks of treatment after PD diagnosis, all three investigated doses of levodopa (150, 300, and 600 mg/d) improved parkinsonism compared to placebo in a dose-dependent manner, but that the highest dose caused side effects like dyskinesia (Fahn et al. 2004).

The American Academy of Neurology and the National Institute for Health and Care Excellence guidelines emphasize levodopa's superior symptomatic efficacy over DA agonists, recommending levodopa as the initial treatment for PD (Rogers et al. 2017; Pringsheim et al. 2021). However, DA agonists or MAO-B inhibitors may be more appropriate in patients with dyskinesia risk factors, or can be proscribed based on the patient’s preferences (Pringsheim et al. 2021).

The Levodopa in Early Parkinson's Disease (LEAP) study was dedicated to comparing long-term effects in PD patients who were treated with levodopa or placebo the first 40 weeks after PD diagnosis, after which they all were treated with levodopa (Verschuur et al. 2019; Frequin et al. 2023; Frequin et al. 2024). Although they did show the expected differences in parkinsonism during those first 40 weeks of differential treatment (Verschuur et al. 2019; Frequin et al. 2023), at 80 weeks no differences in Unified Parkinson’s Disease Rating Scale (UPDRS) scores were observed (Verschuur et al. 2019). In a separate paper though, the same researchers explained that at 80 weeks the patient groups differed somewhat in answering to the binary (YES/NO) questions “Do you notice improvement of symptoms in response to the morning medication?” and “Do the Parkinson's symptoms worsen if you skip or forget the medication?” with the YES/NO ratios to these two questions in the “first-placebo” group being 51/160 and 40/171, respectively, and in the “first-levodopa” group 30/176 and 24/182. The authors were aware of this when writing the Verschuur et al. 2019 paper, but decided not to mention it because, as they explain: “ *We did not present the results of these questions in the original LEAP publication because these questions had not been subjected to a rigorous validation process and because of space limitations*” (Frequin et al. 2023). Regardless, at 80 weeks they did not find a disadvantage of early levodopa treatment (Verschuur et al. 2019; Frequin et al. 2023), and when they followed the same groups until 3 and 5 years after onset, no differences were observed in disease progression or the prevalence of motor complications (Frequin et al. 2024).

The PD Med Collaborative Group compared levodopa treatment (mean daily doses at 1 and 7 years were 347 and 531 mg/day, respectively) with levodopa-sparing treatment, using dopamine agonists or MAOBI (PD Med Collaborative Group et al. 2014). They concluded that the levodopa treatment increased the risk of dyskinesia but that this was not cumulative over time, while also not finding any other cumulative adverse effect of levodopa therapy or a loss of its benefits with time. Overall, they concluded that the levodopa treatment gave the best improvement in mobility scores and that the overall balance of benefits and risks favours levodopa over levodopa-sparing therapy with better patient-rated quality of life both in the short and long term.

As for side effects, when administered for a sufficiently long period of time, levodopa raises plasma homocysteine levels, which are associated with stroke, heart attack, and dementia. Supplementation with Vitamin B12 and folate can counteract this (Foltynie et al. 2024). Increasing dopaminergic replacement therapy has limitations due to levodopa-induced dyskinesia, orthostatic hypotension, impulsive behaviors, and visual hallucinations. However, some of these side effects are more severe when using DA agonists or MAO-BI, and when an increase in dopaminergic therapy is needed, a careful consideration should be made between the different therapeutic options and their possible combinations (De Bie et al. 2020). Of note, regarding the dose it is emphasised to consider the gender as well as the weight of the patient.

### Levodopa therapy during the late PD stage

As the disease progresses, motor fluctuations can occur, such as early morning OFF symptoms, end-of-dose OFF symptoms, dose failures, and delayed ON-time in patients who initially respond well to levodopa (Quinn et al. 1984; De Bie et al. 2020; Livingston and Monroe-Duprey 2024). With the decreasing natural striatal DA, patients require higher doses of levodopa, often in combination with other dopaminergic management. In patients with severe recurrent OFF periods, levodopa may even need to be administered up to every 2 h during the waking day (occasionally with additional doses during the night), with a daily total in the range of 2000 mg (Salat and Tolosa 2013).

Ensuring good compliance with treatment is essential, and physicians should be aware that levodopa absorption can be delayed by slow gastric emptying and blocked by protein intake (Warnecke et al. 2022). Increasing the monotherapy dosage or adjunctive medication is required if the patient does not show improvement, irrespective of such correspondence. Guidelines support the use of DA agonists, COMT-Is, or MAO-BIs to reduce the OFF-time in patients with motor fluctuations. An extended-release oral formulation of levodopa, IPX066, can reduce the OFF-time by approximately 1 h per day compared to immediate-release levodopa but requires careful dose conversion (Hauser et al. 2013). Eventually, also Therapeutic Drug Monitoring (TDM) may help to optimize drug regimens (Hiemke et al 2018; Müller et al. 2024).

Levodopa–carbidopa intestinal gel (LCIG; “Duodopa” or “Duopa”), delivered via an intrajejunal percutaneous tube, has been an effective treatment for severe motor complications (Nyholm et al. 2003; Senek et al. 2017; Antonini et al. 2021). In a systematic literature review, which analysed 27 studies, a stratified analysis of OFF-time in PD patients treated with Duodopa^®^ demonstrated mean relative reductions of 47–82% at 3–6 months and up to 83% reduction at 3–5 years of follow-up (Antonini et al. 2021). However, gastrojejunostomy-related complications, such as tube kinking, dislocation, buried bumper syndrome, and infection, are commonly observed. Furthermore, peripheral neuropathy is a rare but severe complication with an unclear cause, and prophylactic supplementation with vitamins B1, B6, B12, and folate is recommended to mitigate this risk. Diphasic dyskinesias (Tolosa and Compta 2006), which often occur when the pump is stopped at night, are another significant side effect (Marano et al. 2019).

Recently, continuous subcutaneous infusion with foslevodopa-foscarbidopa was introduced (Soileau et al. 2022), representing one of the attempts to retain the benefits of the intrajejunal Duodopa^®^ system while reducing risk and discomfort.

In summary, levodopa is widely used to improve the motor symptoms of PD, from early to advanced stages, with varying methods of administration, routes of administration, and prevention of degradation.

## How levodopa treatment can increase striatal DA concentrations: involvement of the serotonergic system

### Entrance of levodopa across cell membranes at the intestinal lumen and BBB

Levodopa structurally resembles a large neutral amino acid (LNAA; i.e., phenylalanine, tyrosine, tryptophan, leucine, isoleucine, valine, and methionine) and is transported across cell membranes by LNAA transporter complexes. One of the important molecules for the uptake across intestinal enterocytes is L-type amino acid transporter 2 (LAT2) (Camargo et al. 2014), while in the uptake across the endothelial cells of the BBB an important role is played by LAT1 (Kageyama et al. 2000), also known as SLC7A5. LAT1 is a Na^+^-independent, facilitated transporter that is expressed on the luminal side of the endothelial cells of the blood–brain barrier (Ohtsuki and Terasaki 2007). Levodopa competes at these uptake sites with other LNAAs which is especially relevant after the intake of meals rich in proteins (meat, fish, eggs, beans). Therefore, clinicians often prefer administering levodopa 30 min before or 60–90 min after meals are taken, to avoid interference with levodopa uptake.

### Neuron populations with AADC and VMAT2 molecules: in catecholaminergic neurons, but not in 5HT neurons, levodopa and DA are subject to autoregulation

It is assumed that levodopa enters most types of cells through amino acid transporter complexes (Reed et al. 2012; Navailles et al. 2013; Mosharov et al. 2015), but, to the best of our knowledge, there has been little investigation on how levodopa enters neurons. Intracellularly, the critical enzyme for converting levodopa into DA is AADC (Hwu et al. 2013), while the transporter molecule VMAT2 is critical for incorporating DA into vesicles that can deliver DA to the extracellular space (Sulzer et al. 2000; Kannari et al. 2001). In the brain, AADC and VMAT2 are co-expressed in 5HT neurons as well as in the three types of catecholaminergic (CA) neurons: DA neurons, norepinephrine (NE) neurons, and epinephrine (E) neurons. In the CA neurons, the conversion of levodopa imported from the extracellular space must compete with endogenous levodopa and DA, and is subject to molecule-matching cell-type specific regulatory mechanisms (“autoregulation”). In contrast, 5HT neurons convert tryptophan to 5-hydroxytryptophan (5HTP), which is then decarboxylated by AADC to form 5-hydroxytryptamine (5HT or “serotonin”), which is subsequently packaged into vesicles by VMAT2. When levodopa enters 5HT cells, it uses these molecules for being converted to DA that is packed together with 5HT molecules in vesicles and then co-released as an assumed (so-called) “false neurotransmitter” (Ng et al. 1970; Muñoz et al. 2020). Therefore, while levodopa treatment of 5HT neurons is theoretically expected to always increase DA release—since these cells do not naturally have levodopa or DA—levodopa treatment of CA neurons is less predictable and depends on the specific limiting factors for DA production and release (autoregulation) in those cells. Nevertheless, many researchers appear to instinctively assume that most of the increased DA production in the striatum resulting from levodopa treatment, at least during early PD, derives from levodopa-to-DA conversion in the axons of SNc DA neurons (e.g., Carta et al. 2008; see also Melamed et al. 1980a). However, while the evidence for a role of 5HT neurons in extra DA production in the striatum is convincing, the evidence for a similar role of DA neurons—to the best of our knowledge—remains only suggestive. It is well proven, though, that the SNc DA neurons play an important role in preventing the striatal extracellular DA concentrations from rising too high after levodopa administration.

### Do SNc DA neurons use levodopa to increase extracellular striatal DA concentrations? Maybe not, but they help control these concentrations

This paragraph describes some studies suggesting a role for SNc DA neurons in the upregulation of striatal DA after levodopa treatment, as well as some studies arguing against such a role.

*Suggestive for.* An important study was performed by Ito et al. (1999), using rats in which only the right SNc was treated with the toxin 6-OHDA that kills CA neurons. They found that one hour after in vivo intraperitoneal injection of radioactive levodopa, after sacrificing the rat, in coronal brain sections including the striatum most radioactivity was found by far in the intact left striatum, implying that most intracellular radioactive levodopa or its derived radioactive DA was present in the remaining SNc DA neurons. However, they also found some radioactivity in the right striatum, which probably could be explained by its incorporation into 5HT neurons. While this study is very suggestive of SNc DA neurons in the striatum taking up radioactive levodopa and converting it to DA, two theoretical possibilities probably cannot be excluded: (i) the increase of radioactive levodopa and/or its derived DA does not necessarily mean that the total amount of DA molecules has increased in SNc DA cells, because regulatory mechanisms may have reduced the production of DA from endogenous levodopa, and (ii) the radioactivity in the SNc DA cells probably was at least partially derived from radioactive DA secreted by 5HT neurons and then taken up by SNc DA neurons through their DAT transporters.

*Suggestive for.* Another study providing very suggestive evidence that levodopa treatment can substantially increase the DA production in SNc DA neuron cell bodies is from Mosharov et al. (2009). These researchers used intracellular patch electrochemistry to measure DA concentrations in the cell bodies of cultured rat TK^+^ midbrain neurons, enriched in SNc neurons. However, it should be considered that: (i) they measured DA concentrations and not DA production, and theoretically the DA might have been taken up after being produced from levodopa by other cells, and (ii) in this out-of-tissue condition the natural mechanisms for producing endogenous DA and controlling DA levels might not be in place.

*Arguing against.* In clinical settings, in contrast to the above animal experiments, evidence suggests that in patients levodopa treatment does not enhance DA production by their SNc DA neurons. Namely, high intracellular DA levels have been suggested as one of the major causes of neuromelanin production and SNc DA neuron vulnerability to PD (Zucca et al. 2023), but levodopa treatment leads neither to increased production of neuromelanin (Mann and Yates 1983; Nagatsu et al. 2023) nor to increased SNc DA neuron degeneration (reviewed in Simuni and Stern 1999; Fahn 2006).

*Arguing against.* That after levodopa administration the SNc DA neurons predominantly have a regulatory/buffering function rather than help to increase striatal DA levels is strongly suggested by high doses of levodopa, up to 100 mg/kg, not causing an increase in DA in intact striatum in rat (Navailles et al. 2010).

*Arguing against.* A study by Abercrombie et al. (1990) is also inconsistent with the importance of SNc DA neurons in increasing striatal DA concentrations after levodopa treatment. Using microdialysis, they found in rats that the DA concentration of striatal extracellular fluid after levodopa treatment was several-fold higher in brains or hemispheres in which SNc DA neurons had been eliminated by 6-OHDA, thus mirroring the final stage of PD. The authors showed that treatment with levodopa caused similarly high DA increases in intact rats if given the drug nomifensine, probably due to its inhibition of DAT, thereby preventing the SNc DA neurons from regulating the DA concentration in the extracellular space through DA uptake. These findings were confirmed in other studies (Miller and Abercrombie 1999; Kannari et al. 2000). The buffering effect of SNc DA neurons, involving the uptake of excess extracellular DA through their DAT transporters, can also explain why for eliciting behavioral effects in healthy animals much higher doses of levodopa are required than in animal PD models (Schoenfeld and Uretsky 1973; Hollister et al. 1979).

*Summary.* In short, levodopa-derived DA does end up in SNc DA neurons. However, because of their autoregulatory capacity dedicated to control DA production, it is questionable whether these cells directly contribute to the enhanced extracellular striatal DA concentration induced by levodopa treatment in PD. In contrast, there is strong evidence that these cells help regulate striatal DA levels and prevent them from becoming too high under normal physiological conditions as well as in the early stage of PD.

### 5HT neurons are major contributors to the increase in striatal DA after levodopa treatment

The involvement of 5HT neurons in the conversion of levodopa to DA, which if SNc DA neurons are depleted can result in large increases in striatal DA concentration, is supported by compelling evidence (reviewed by Chagraoui et al. 2019).

For example, in the striatum of a rat 6-OHDA (late/final stage) PD model, treatment with the serotonergic neurotoxin 5,7-dihydroxytryptamine (5,7-DHT) caused a 79% decrease in the striatal extracellular DA concentration induced by levodopa treatment (Tanaka et al. 1999). Similar effects of 5HT neuron depletion were found in other studies (Lopez et al. 2001; Carta et al. 2007). However, in a rat model with intact SNc DA neurons, no contribution of 5HT neurons to levodopa sensitivity was observed (Hollister et al. 1979), implying that in the intact striatum the regulatory effects of the SNc DA neurons override this potential of 5HT neurons.

Treatment with tetrodotoxin (TTX), an inhibitor of fast sodium channels, inhibited the upregulation of extracellular striatal DA concentrations by levodopa treatment in 6-OHDA PD rats, suggesting that 5HT neurons need to be firing to enhance DA production (Miller and Abercrombie 1999). In accordance, reductions in the increase in extracellular DA in the striatum after levodopa administration were also found if reagents were used that more specifically inhibit 5HT neuron firing, such as 8-OH-DPAT, an agonist of the inhibitory serotonin autoreceptor 5-HT1A (Kannari et al. 2001) and fluoxetine, a selective serotonin reuptake inhibitor (SSRI) (Yamato et al. 2001).

The DA-producing capacity of 5HT neurons in the striatum, upon levodopa treatment, was also supported by immunohistochemistry in normal rats (Arai et al. 1995) as well as in the 6-OHDA PD model (Yamada et al. 2007).

On a note, in rats depleted for both SNc DA and 5HT neurons, extremely high levodopa administrations (100 mg/kg, but not 30 mg/kg, and compared to 1–6 mg/kg being common in the clinic) could still increase striatal DA concentrations and elicit DA-induced behaviour (Melamed et al. 1980a; Hollister et al. 1979; Lopez et al. 2001; Navailles et al. 2010; reviewed by Navailles et al. 2013). This might be explained by levodopa being metabolised to DA via AADC in other cell types, e.g., astrocytes and capillaries, thus acting in part in a hormone-like manner at striatal DA-receptors.

In summary, at least when the number of SNc DA neurons is substantially diminished, like in late/final stages of PD, the 5HT neurons are the dominant population for converting administered levodopa into DA and increasing extracellular striatal DA. The 5HT neurons release their levodopa-derived DA based on the 5HT firing pattern, which is regulated by the serotonergic system as discussed below—therefore, it is said to be released as a “false neurotransmitter” or that its release is “uncontrolled.”

### Natural functions of the serotonergic system: implications for levodopa-derived DA release, and the effects of PD and levodopa treatment on 5HT release

The levodopa-induced release of DA by 5HT neurons is based on the anatomy and signalling of the serotonergic system, involving timing and locations different from normal DA release from intact SNc DA neurons. This not only changes the DA release pattern, but also decreases 5HT release due to apparent pathway competition within 5HT neurons between the two neurotransmitters.

#### Distribution, signalling, and function of the serotonergic system

The serotoninergic network in the brain originates in the raphe nuclei of the brainstem and projects to most brain regions (reviewed in Hornung 2003; Hensler 2006; Pourhamzeh et al. 2022). These nuclei include the dorsal raphe, raphe magnus, median raphe, raphe obscurus, and raphe pontis. It is primarily the dorsal and median raphe nuclei that project to the forebrain areas. The raphe nuclei receive signals from the hypothalamus, cortex, and limbic forebrain structures, including the amygdala (for details see Stansley and Yamamoto 2015).

5HT is a neurotransmitter that modulates a wide range of functions, including mood, perception, appetite, aggression, anxiety, reward processing, cognition, memory, learning, sexuality, attention, respiratory stability, sleep–wake cycles, body temperature, and circadian rhythms. The striatum is important for learning, and 5HT-induced activity in the striatum was found associated with delayed reward prediction (Tanaka et al. 2007).

The release of 5HT is mainly based on volume transmission, and varicose axons from the dorsal raphe nucleus (DRN) branch profusely in their target areas, including the striatum (reviewed by Hensler 2006). Most of the 5HT neurons in the DRN fire tonically at a low rate during waking, progressively slower during slow-wave sleep, and stop firing during rapid eye movement (REM) (Sakai and Crochet 2001). The activity of DRN 5HT neurons can be increased by mediators of arousal such as orexin/hypocretin, histamine, or NE (Brown et al. 2002). Transient activation by optogenetics of DRN serotonin neurons caused brain-wide activation, including the medial prefrontal cortex, the striatum, and the VTA (Hamada et al. 2024).

Many questions remain about the mechanisms of serotonergic control of brain activities, and, for example, it is not well understood at a detailed level why selective serotonin re-uptake inhibitors (SSRIs)—which increase extracellular 5HT concentrations—help to treat various psychiatric disorders (Edinoff et al. 2021).

#### How is DA released differently by the serotonergic system?

Neither the dopaminergic nor the serotonergic systems are understood in great detail, and many questions remain unanswered. However, some general statements can be made.

*Lack of autoregulation.* Release of levodopa-derived DA by 5HT neurons is not controlled by proper autoregulation processes, and extracellular DA is not taken up by 5HT neurons as they have no DAT molecules (Muñoz et al. 2020). This means that in the late PD striatum, when the buffering/controlling capacity of SNc DA neurons is diminished, levodopa treatment can generate large fluctuations in DA concentration (Fig. 2).

*Less extensive innervation of the striatum.* In the dorsomedial and dorsolateral striatum of healthy rats, tissue DA concentrations are > 40 times higher than 5HT concentrations (Fitoussi et al. 2013). Fitoussi et al. (2013) concluded that these neurotransmitter concentrations correlated with different levels of neuron innervation, based on comparing their results with several prior studies that investigated either DA neuron innervation or 5HT neuron innervation.

*Distribution to different sites.* 5HT is delivered to most brain regions (Hensler 2006), so levodopa-derived DA is also expected to be broadly distributed. In accordance, after levodopa treatment of 6-OHDA rats mirroring the late/final stage of PD, high levels of extracellular levodopa-derived DA were found not only in the striatum but also in the SNr, hippocampus, and prefrontal cortex (Navailles et al. 2010; 2011). These DA increases required the activity of 5HT neurons (Navailles et al. 2010; 2011). Thus, it can be concluded that the release of levodopa-derived DA via 5HT neurons creates a new DA chemistry throughout the parkinsonian brain (Navailles and De Deurwaerdère 2011).

Within the healthy striatum, the innervation pattern of the DA neurons and 5HT neurons is also different. Especially within younger people, in the putamen the DA levels increase from rostral to caudal, whereas for the 5HT concentration an opposite trend was observed (Hörtnagl et al. 2020).

*Different timing of release.* The striatal DA and 5HT systems are both more quiescent during sleep than when awake. A difference is that during REM sleep the intact striatal DA system can be quite active—a lack of which may help explain why many PD patients have sleep disorders (Lima 2013)—whereas the striatal 5HT system is inactive (Sakai and Crochet 2001). However, most PD patients are not treated with levodopa during sleep, thus generally this difference will have little impact on levodopa-derived DA release. During the wake phase, both the striatal DA and 5HT systems are characterized by continuous firing, providing a tonic background level of neurotransmitter that can be temporarily altered by specific signals that change the firing rate. To which extent the specific signals for the dopaminergic and serotonergic systems coincide or not is not well known and beyond the scope of this review. They do not fully coincide though (Cools et al. 2011), and thus, after levodopa administration, the 5HT system cannot fully restore the functions of the healthy DA system in case many SNc DA neurons died.

Of note, the firing rate of DRN 5HT neurons is lower than that of SNc DA neurons (⁓1.5 Hz vs. ⁓4.5 Hz) (Harden and Grace 1995; Miguelez et al. 2016).

#### Reduced 5HT production because of levodopa treatment

In 5HT neurons the levodopa-derived DA uses a similar production and release pathway, although not properly controlled, as the endogenous 5HT, involving AADC and VMAT2. This seems to lead to competition, as in the 6-OHDA rat PD model the treatment with levodopa led to a 48% decrease in striatal serotonin (Carta et al. 2008). Others reported similar findings in 6-OHDA rats treated with levodopa not only for the striatum, but also showed that in the SNr, hippocampus, and prefrontal cortex the 5HT concentrations were decreased (Borah and Mohanakumar 2007; Navailles et al. 2010; Navailles and De Deurwaerdère 2011). These findings were consistent with earlier findings of decreases in rat whole brain 5HT after levodopa treatment (Bartholini et al. 1968; Uretsky and Schoenfeld 1971). This decrease in rat whole brain 5HT was independent of DA neuron inactivation, at least under high doses of levodopa of ≥ 100 mg/kg (Uretsky and Schoenfeld 1971). Levodopa was also found to have various other biochemical, morphological, and molecular impacts on the serotonergic system, which is only logical, as levodopa reduces tryptophan transport across the BBB and—after cellular uptake—directly affects the metabolism of 5HT cells (reviewed in Navailles and De Deurwaerdère 2011; Reed et al. 2012).

#### Gradual decrease of the serotonergic system during PD progression

The serotonergic system tends to gradually degenerate during PD progression. However, its level of degeneration can differ considerably between patients and is less pronounced than that of the dopaminergic system (Jellinger 1991; 2019). It is also difficult to distinguish in many reports to which extent the degeneration of the serotonergic system is intrinsic to PD or may represent the effects of levodopa treatment.

In PD patients, a tendency was observed toward lower neuron densities in the DRN, especially in those with depression (Paulus and Jellinger 1991). However, the range of these neuron densities substantially overlapped with that found for healthy controls (Halliday et al. 1990; Paulus and Jellinger 1991). Braak et al. (2003a, b) found that Lewy bodies in the raphe nuclei are rather common already early during PD progression (from “stage 2”), suggesting a direct impact of the disease on the serotonergic system. From the 1980s, “functional damage” to the serotonergic system in PD was concluded based on decreases in concentrations of 5HT and its receptors (reviewed by Jellinger 1991).

Kish et al. (2008) studied brain autopsies from a group of 23 deceased patients who on average were diagnosed with PD ⁓14 years earlier. In this group, on average, the DA concentrations of the caudate and putamen decreased by 80% and 98%, respectively, compared to age-matched controls. In the same study, a comparison between the average 5HT concentrations in the caudate and putamen showed reductions in the PD group of 66% and 51%, respectively, and reductions in other serotonergic markers were found as well (Kish et al. 2008).

Pagano et al. (2017a) performed a meta-analysis of 20 studies in which PET was used for serotonin transporter (SERT) imaging, in order to study the correlation between PD, SERT distribution, and symptoms. SERT is expressed by 5HT neurons for reuptake of secreted 5HT, and a marker of the serotonergic system. The meta-analysis indicated that, on average, SERT detection was reduced between 27 and 42% in the PD patients, differing per brain region: hypothalamus, − 27%; caudal raphe, − 28%; ventral striatum, − 29%; putamen, − 31%; thalamus, − 32%; rostral raphe, − 41%; and caudate, − 43%. This meta-analysis concluded only for the putamen a statistically significant correlation between these decreases and disease duration, which is puzzling but may be related to the difficulty of analysing multiple different studies together.

In many PD patients, a continuing degeneration of 5HT supply is believed to exacerbate both motor and non-motor symptoms (Politis and Niccolini 2015; Pagano et al. 2018).

## The norepinephrine (NE, also known as noradrenaline) system in PD

The locus coeruleus (LC) with its NE neuron cell bodies is already affected very early in the pathogenesis of PD, and degeneration of the LC is observed even before that of the SNc (Braak et al. 2003a, 2003b; Vermeiren and De Deyn 2017). In different studies, reductions in the neural densities in the LC of PD patients were reported between 28 and 88%, with the higher values positively correlated with dementia (reviewed in Jellinger 1991; Paredes-Rodriguez et al. 2020). This degeneration reduces the noradrenergic input into the striatal system, as well as the cortical and limbic brain areas (Ehringer and Hornykiewicz 1960; Gibb and Lees 1991; Goldstein et al. 2011). In a group of PD patients with an average disease duration of 12 years and an average loss in DA concentration in the caudate nucleus of > 80%, the average NE loss in the frontal cortex was ⁓75% (Scatton et al. 1983). In another study, in the PD striatum, in a group of patients that on average showed ~ 72% loss of DA content, only reductions of ~ 29% in NE concentration were observed (Ehringer and Hornykiewicz 1960). In summary, NE losses in PD can be severe, but are on average not as pronounced as nigrostriatal DA losses.

In NE neurons, levodopa is metabolized by AADC to DA, which is further converted to NE by dopamine-ß-hydroxylase. In the parkinsonian LC, cellular concentrations of neuromelanin—a melanin aggregate eventually accumulating in association with high DA or NE production—are positively associated with NE neuron degeneration, underlining the toxicity of the DA/NE synthesis pathway (reviewed by Nagatsu et al. 2023). Theoretically, levodopa treatment might lead to an increase in NE production by NE neurons since levodopa is a substrate for NE synthesis. Arai et al. (2008) have provided evidence that in the 6-OHDA PD model levodopa-derived DA is taken up in the striatum by the NE-transporter. However, there is no evidence from clinical studies or postmortem analyses of levodopa treatment having such an effect in PD patients, or that NE neurons increase their NE release based on levodopa-derived NE production.

LC NE neurons in the healthy brain can co-release DA to several brain regions, and in the dorsal hippocampus this DA release is believed to be important for spatial learning and memory (Devoto and Flore 2006; Kempadoo et al. 2016).

The deterioration of the noradrenergic system in PD and the resulting reduction in NE and DA is believed to cause and exacerbate non-motor symptomatology, like depression, anxiety, anhedonia, apathy, or fatigue (Birkmayer and Riederer 1986; Jellinger and Paulus 1992; Herlofson and Larsen 2002; Pavese et al. 2010; Lima 2013; Grosch et al. 2016; Kohl and Winkler 2020) and also promote neuroinflammation that may contribute to PD progression (reviewed in Paredes-Rodriguez et al. 2020).

In summary, while in theory levodopa administration might have a significant effect through uptake by NE neurons and release as DA or extra NE, there appears to be no evidence for that. However, the PD-induced degeneration of the NE system can have a profound effect on the disease phenotype.

## Short-duration responses (SDR) and long-duration responses (LDR): the effects of neural plasticity and priming

Levodopa exerts its therapeutic effect on PD through two mechanisms of action: short-duration responses (SDR) and long-duration responses (LDR). SDR to levodopa refers to the immediate effect lasting a few hours and is closely associated with its plasma pharmacokinetics. LDR to chronic use of levodopa develops over days to weeks and lasts beyond drug elimination, with its effects only decaying slowly (Kang et al. 2012; Zhang et al. 2013b; Zhuang et al. 2013). LDR is a sensitization response to levodopa treatment, often but not always beneficial, and believed to involve corticostriatal neural plasticity (Zhuang et al. 2013). As discussed in the previous chapters of this review, levodopa treatment induces DA delivery by 5HT neurons, which involves different sites and timing compared to endogenous DA delivery. The striatum is programmed for learning, and this function includes the adaptation of its neural circuits, importantly through neuroplasticity of MSNs, to the strengths and locations of incoming signals, among which DA is an important one. This adaptation, although the detailed features are not known, is believed to cause the LDR effect. The adaptive effects of previous levodopa administration can also be called “priming,” but that word tends to be more narrowly used in connection to the promotion of LID (Nadjar et al. 2009).

A recent study calculated that LDR contributes 60–65% of the total motor benefit from levodopa, independent of disease duration (Cilia et al. 2020). Although two-thirds of patients developed diurnal motor fluctuations within 1 year, OFF-state motor performance was consistently better than the pretreatment baseline, and a sustained LDR was observed even after 7 days of withdrawal (Cilia et al. 2020).

Kang et al. 2012, compared the effects of learning a finger-tapping task between levodopa-treated and placebo-treated PD patients and found that the levodopa treatment led to significantly improved task performance. This improved performance was sustained even two weeks after levodopa was withheld following 40 weeks of levodopa treatment. The experiment provided evidence for the overlap between the LDR to levodopa and normal motor learning functions.

Brain MRI and neurophysiological studies support the view that LDR may be related to adaptive changes in neuroplasticity in basal ganglia and cortical networks (Donzuso et al. 2021; Alhassen et al. 2024).

## Levodopa-induced side effects during late PD: OFF-phase, levodopa-induced dyskinesia (LID), and other problems

Building on the discussions from earlier chapters, this chapter focuses on OFF-phase and LID issues in late PD. These complications are primarily caused by the continuing alterations of the dopaminergic system and the larger fluctuations in striatal extracellular DA concentrations following pulsatile levodopa treatment. However, individual differences in sensitivity (including the 5HT system between patients) to these fluctuations probably also affect outcome. Furthermore, although not commonly agreed, it has been postulated that chronic levodopa treatment may lead to toxic side effects.

### The major reasons for OFF-phase and LID issues: degeneration of DA neurons and fluctuations in striatal extracellular DA through pulsatile stimulation with levodopa

Levodopa treatment does not necessarily lead to OFF-phase and LID issues, as these complications tend to be absent during the early phase of PD and during the lifelong treatment of Segawa disease (see earlier in this review). As discussed above, the key difference between these conditions and late-stage PD is the ongoing decline of the dopaminergic system, including the loss of its DA buffering capacity. This causes an increase of the relative impact of the levodopa-derived DA release mode by 5HT neurons. As a result, fluctuations in striatal extracellular DA concentrations become more pronounced, following the pulsatile administration pattern of levodopa with higher peaks (increasing the risk of LID) and deeper troughs (creating OFF-phases) (Fig. 2).

That LID is caused, in late PD, by larger changes in striatal DA levels following levodopa administration, has been implicated by PET imaging studies (e.g., de la Fuente-Fernandez et al. 2004; Pavese et al. 2006). De la Fuente-Fernandez et al. (2004) compared PD patients with and without LID symptoms and found that, in the LID group, 1 h after an identical dose of levodopa the availability of dopamine receptors for binding of [^11^C]raclopride (a measure inversely correlated with the extracellular DA concentration) had decreased by 16% and 17% in the caudate nucleus and putamen, respectively, while only 7% and 9% in the non-LID group. These findings were confirmed, in similar experiments, by Pavese et al. (2006).

The association between LID and high DA concentrations was also indicated by Politis et al. (2014), who found that identical levodopa doses induced markedly higher striatal extracellular DA concentrations in PD patients with LIDs compared with PD patients with stable responses to levodopa.

The above findings for human PD were supported by studies in rodent models (e.g., Meissner et al. 2006; Lee et al. 2008). Meissner et al. 2006 found in a rat 6-OHDA PD model that repeated levodopa treatment caused much higher striatal extracellular DA concentrations than the levels found in untreated control rats and that these high concentrations induced abnormal involuntary movements (AIMs, the rodent equivalent of LID). Lee et al. (2008) found that in rats the denervation of the dopaminergic system caused levodopa treatment to induce unusually high striatal extracellular DA concentrations associated with AIMs. However, in contrast to the report by Meissner et al. (2006), in the Lee et al. (2008) study this effect did not depend quantitatively on earlier treatments with levodopa.

### Studies suggest that DA levels are not the only difference between individuals with and without LID symptoms: possible impacts of variation in the 5HT system

#### Studies suggesting not only DA levels are important for LID

PD not only affects DA neurons, but also other parts of the nervous system, and patterns of disease progression in individual patients can be so different that PD subtypes have been distinguished (Riederer et al. 2023). Furthermore, even independent of PD, it appears reasonable to assume some natural differences between individuals in their brain’s plastic ability to adapt to low DA concentrations as well as to levodopa-derived DA being supplied by 5HT neurons (for both topics see earlier chapters). This agrees with the generation of LID not showing a perfect correlation with the estimated striatal extracellular DA concentrations (Pavese et al. 2006). Notably, PD progression is a complicated, human-specific process, which makes it difficult to study, and most of the proposed factors for explaining LID differences between PD patients appear to need further discussion.

Animal PD models are substantially different from human sporadic PD in the time required to develop and the underlying pathologies. Nevertheless, some animal studies seem very informative in the context of this paragraph. Bézard et al. (2001a, b; 2003) found in a monkey 1-methyl-4-phenyl-1,2,3,6-tetrahydropyridine (MPTP) PD model that despite similar levels of MPTP-induced damage and identical levodopa treatment only some of the individual monkeys showed LID. Lindgren et al. (2010), using a rat 6-OHDA PD model, reported that the variations between individuals in their striatal extracellular DA concentrations after chronic treatment with levodopa did not predict well whether they exhibited AIMs. Therefore, the authors concluded that both a high release of levodopa-derived DA and an increased responsiveness to DA must coexist for a full expression of dyskinesia (Lindgren et al. 2010).

#### Possible effects of 5HT neuron variation on LID: controversies in the field

Because the 5HT system appears to supply most of the striatal DA in late PD as an assumed (so-called) “false” neurotransmitter derived from levodopa (see earlier in this review), differences in this system are expected to affect OFF-phase and LID occurrences. However, there are conflicting studies on this matter, so we hope the reader can bear with us that this paragraph is quite complicated.

Some studies discuss that a high ratio between the 5HT and DA systems may promote LID (Pagano et al. 2018; di Biase et al. 2023). However, this hypothesis seems predominantly based on the striatal DA system degenerating faster during PD progression than the striatal 5HT system, and LID being a feature of late PD (Lee et al. 2015; Roussakis et al. 2016). Quite interestingly, however, dyskinesia was also experienced by two PD patients who did not receive levodopa treatment but showed excessive serotonergic innervation in their striatum tissue derived from grafted DA-rich fetal mesencephalic tissue. Treatment with the 5HT_1A_ receptor agonist buspirone almost completely resolved their dyskinesia, providing suggestive evidence that even in the absence of levodopa treatment, by an unclarified mechanism, a high ratio of 5HT to DA in the striatum may induce dyskinesia (Politis et al. 2010; 2011).

In the opposite direction, a study by Tronci et al. (2013) might suggest that a high DA to 5HT ratio promotes LID. Namely, using a rat 6-OHDA PD system they found that treatment with 5HTP, the precursor of 5HT, led to a reduction in levodopa-induced AIMs while not reducing the therapeutic effect of levodopa (Tronci et al. 2013). However, that study was probably not sufficiently detailed to exclude the simpler explanation that 5HTP treatment, through promoting 5HT synthesis and pathway competition, reduced DA synthesis by 5HT neurons.

Another study in 6-OHDA PD rats found a positive correlation between levodopa-induced AIMs and the size of the striatal 5HT system (measured by SERT imaging), whereas the size of the remaining striatal DA system (measured by DAT imaging) had no apparent impact (Rylander et al. 2010). Also supporting a central role of 5HT neurons, Beaudoin–Gobert et al. (2015) found that the damaging of 5HT nerve terminals by 3,4-methylenedioxy-N-methamphetamine (MDMA) in the brain of MPTP PD monkeys caused a decrease in LID. Interestingly, those authors also found that independent of levodopa this MDMA treatment increased rigidity in the monkeys. This agreed with their unpublished observation for PD patients that the greater the 5HT denervation in the putamen, the greater the patient’s rigidity (Beaudoin–Gobert et al. 2015).

In rats, it was reported that lesioning of the nigrostriatal DA system using 6-OHDA caused striatal serotonergic axonal hyperinnervation/sprouting (Zhou et al. 1991; Maeda et al. 2003; 2005). However, such findings appear to disagree with other studies showing that 6-OHDA treatment of rats did not induce an increase—and could even cause a decrease—in brain or striatal 5HT concentration (Iwamoto et al. 1976; Breese et al. 1984; Karstaedt et al. 1994).

The latter finding agreed well with a study by Rylander et al. (2010), who found that 6-OHDA treatment caused a significant decrease in the rat striatal 5HT system (measured by [^3^H]citalopram labeling). However—showing a partial overlap with the above-mentioned hyperinnervation reports—the authors found that the treatment of those rats with levodopa induced sprouting of striatal 5HT axon terminals in a manner that correlated positively with the severity of AIMs, despite the fact that the total size of the striatal 5HT system (measured by [^3^H]citalopram labeling) remained below that of the intact striatum. These authors reported very similar findings on the size of the putaminal 5HT system and the effects of levodopa treatment in a monkey MPTP PD model (Rylander et al. 2010). Furthermore, by analysis of post-mortem brains, they found a similar situation in humans, showing that human PD was associated with a decreased putaminal 5HT system and that higher LID values were associated with larger sizes of the putaminal 5HT system (measured by [^3^H]citalopram labeling). However, in the absence of negative control samples, they could not estimate the effect of levodopa treatment in human PD patients (Rylander et al. 2010). The development of human PD is very different from these animal models, thus extrapolation of findings—like the enhanced sprouting—should be done with care.

Unlike Rylander et al. (2010), the Politis et al. (2014) study did not find a correlation in PD patients between the sizes of their striatal 5HT system (measured by ^11^C-DASB PET) and their LID profiles. This is also consistent with reports finding no correlations in PD patients between their LID profiles and the concentration of striatal 5HT (Calon et al. 2003; Kish et al. 2008; Cheshire et al. 2015), striatal SERT, or the number of 5HT neurons in the dorsal raphe nuclei (Cheshire et al. 2015).

In short, there are considerable differences between individual PD patients in their striatal 5HT systems. However, how these differences originate, and impact PD progression and levodopa treatment, remains a topic of ongoing debate.

### Impacts on LID of other neural populations besides DA and 5HT neurons

While the modulation of especially dopaminergic and serotoninergic functions in the striatum are of major interest when discussing LID, also the noradrenergic, gluatamatergic, adenosinergic, opioid-related, and even other systems have been proposed to affect the development of these motor complications (Pagano et al. 2018).

#### The noradrenergic system and LID

For some more general aspects of the noradrenergic system in PD, see the earlier chapter on this topic.

Experimental studies in rats provided evidence that the noradrenergic system is involved in LID (Fulceri et al. 2007; Buck and Ferger 2009; Miguelez et al. 2011), but this is not without discussion as reported in detail by Cenci (2014). Pharmacological studies supported the hypothesis that NE can be involved in generating LID because DA can be taken up by the NE transporter in experimental rodents (Arai et al. 2008; Nishijima and Tomiyama 2016) and several different adrenoceptor antagonists alleviated dyskinetic movements induced by levodopa in 6-OHDA rats (Buck and Ferger 2010; Buck et al. 2010). The experimental study by Wang et al. (2014) compared NE release in the ipsilateral sensorimotor striatum of dyskinetic and nondyskinetic 6-OHDA-lesioned hemiparkinsonian rats treated chronically with levodopa. Intrastriatal perfusion of NE into the lesioned sensorimotor striatum induced a moderate dyskinesia in dyskinetic rats, which was similar to the dyskinetic behavior after levodopa administration. The levodopa-related dyskinetic behavior was inhibited significantly by a further pretreatment of noradrenergic neurotoxin N-​(2-​chloroethyl)​-​N-​ethyl-​2-​bromobenzylamine or intrastriatal administration of the α_2_-adrenoceptor antagonist idazoxan. Therefore, abnormal NE neurotransmission has been suggested to contribute to the induction of LID (Wang et al. 2014).

#### Medium spiny neurons (MSNs) and LID

If striatal DA concentrations change, MSNs appear to change their circuits and adapt their thresholds for D1 and D2 receptor signalling. However, there is considerable debate on the precise mechanisms (see earlier). Therefore, although it is only logical that this MSN plasticity affects the occurrences of OFF-phase and LID, we will mostly refrain here from a further discussion on this topic and refer to earlier parts of this review and other studies (Gerfen et al. 1995; Muriel et al. 1999; Bézard et al. 2001a, b; 2003; Wahyu et al. 2021). The above paragraph "Other neural systems and LID" includes some discussion of the changing input that the MSNs receive from different neural populations.

#### Other neural systems and LID

Several findings support the hypothesis that glutamatergic transmission plays a significant role in LID pathogenesis (Campanelli et al. 2022).

Glutamatergic projections from the corticostriatal region enter the putamen, where glutamate interacts with ionotropic (NMDA and AMPA receptors) and metabotropic mGlu5 receptors on medium spiny neurons. Increased spontaneous glutamate release following DA denervation and elevated glutamate levels after levodopa administration result in glutamate hyperactivity (excitotoxicity) in the striatum of PD patients with LID. Amantadine, an NMDA receptor antagonist that stabilizes the glutamate pathway, effectively reduced dyskinesia (Rascol et al. 2021).

Acetylcholine is crucial in striatal plasticity and LID pathophysiology, modulating DA release through nicotinic acetylcholine receptors on nigrostriatal axons. Cortical and thalamic glutamatergic inputs regulate cholinergic interneurons and influence cholinergic and dopaminergic transmissions in the striatum (Kosillo et al. 2016). Nicotinic receptor agonists have been shown to reduce dyskinesias in rat and monkey models (Quik et al. 2013; Zhang et al. 2013a). In transgenic mice, muscarinic receptors (M1 and M4) affect long-term potentiation (LTP) and long-term depression (LTD) at corticostriatal synapses, with M4 receptor modulation reversing aberrant LTP and reducing dyskinesia (Wang et al. 2006; Shen et al. 2016). Histamine H2 receptors, which are highly expressed in cholinergic interneurons, are also potential targets, as their blockade by famotidine decreases LID in dyskinetic animals (Lim et al. 2015).

Earlier in this review, we discussed “long duration response (LDR),” which concerns brain plasticity-driven adaptations to levodopa treatment that are rather described as “priming” when leading to enhanced LID responses. Although the precise mechanisms are undetermined, imaging and electrophysiological studies showed that network plasticity including the cerebral and cerebellar cortex can be associated with LDR (Donzuso et al. 2021; Sciacca et al. 2023). Brain imaging studies have also shown network abnormalities in the putamen, supplementary motor cortex, inferior frontal cortex, motor cortex, sensory cortex, and cerebellum of patients with LID (Espay et al. 2018).

#### Astrocytes and LID

As AADC is detected in astrocytes (Tsai and Lee 1996; Nakamura et al. 2000) and blood vessel-associated cells (Bertler et al. 1966; Hefti et al. 1980; Melamed et al. 1980b) it has been suggested that levodopa might be metabolized to DA in these cells (Cenci 2014). Then DA has been hypothesized to diffuse in an uncontrolled matter to reach dopaminergic receptors in the striatum, although rather at high levodopa doses (Ruscher et al. 2012). However, because both astrocytes and endothelial cells contain MAO, which rapidly metabolizes levodopa-derived DA, it is unlikely that LID is caused by this mechanism. Nonetheless, effects of levodopa on neurovascular systems and regional blood flow cannot be excluded and await further investigations as reviewed by Cenci (2014).

### Medication to reduce OFF-time

To improve levodopa pharmacokinetic/pharmacodynamic properties as well as side effects and adverse reaction profiles, a number of new galenic forms and types of application have been developed in recent years, including immediate release (e.g., IPX203), extended-release, and soluble levodopa formulations (see Müller et al. 2024). Extended-release carbidopa-levodopa (IPX066) reduces OFF-time without enhancing dyskinesia (LeWitt et al. 2018).

Istradefylline (IST), an adenosine A2A receptor antagonist, has been introduced for PD patients experiencing OFF periods (Cummins and Cates 2022) or the wearing-OFF phenomenon (Takahashi et al. 2022). Additionally, IST reduces levodopa dose escalation (Hattori et al. 2023; Hatano et al. 2024).

Amantadine, when used as an adjunct to levodopa, can markedly improve motor complications (Verhagen Metman et al. 1998), significantly antagonise akinetic crisis (Danielczyk 1973), reduce LID (Pahwa et al. 2015; Dashtipour et al. 2019), and reduce OFF-time in PD patients (Hauser et al. 2022). However, in the ALLAY-LID studies, immediate release/extended-release amantadine (OS320) reduced LID but not OFF-time (Rascol et al. 2022). In contrast, phase III trials of ADS-5102, an extended-release capsule formulation of amantadine, found it beneficial for reducing both dyskinesia and OFF-time in PD (Elmer et al. 2018).

### Rethinking of levodopa long-term toxicity: more controversies in the field

Clinical research has provided conflicting evidence on the long-term toxicity of levodopa in patients with PD. While some studies suggest that levodopa is not toxic beyond its immediate effects (for side effects see earlier in this review) (reviewed by Simuni and Stern 1999; Fahn 2006), even when administered at low doses for many years, others indicate that it may accelerate aging processes via secondary metabolic changes related to decreased methylation capacity (reviewed by Müller and Riederer 2024).

Already above we have mentioned several conflicting reports. The paragraph "Possible effects of 5HT neuron variation on LID: controversies in the field" describes various impressions of how levodopa treatment may or may not affect the 5HT system, while the chapter before that ("The major reasons ...") describes how two similar studies claim that previous levodopa treatments affect (Meissner et al. 2006) or do not affect (Lee et al. 2008) the height of levodopa-induced striatal extracellular DA concentrations.

An important argument against levodopa toxicity is that in a study where levodopa therapy was initiated years after onset of PD, the observed motor fluctuations and dyskinesias were not associated with the duration of levodopa treatment, but with duration of disease and higher dosage of levodopa (Cilia et al. 2014).

Fahn et al. (2004; see also Fahn 1996; 1999; Carlsson 2002; Agid et al. 1998) showed that levodopa versus placebo treatment protected against the severity of PD in patients receiving the diagnosis of PD within the past two years. Also within expectation was that the patients receiving the highest amount of levodopa experienced significantly more dyskinesia, hypertonia, infection, headache, and nausea than those receiving placebo. However, their neuroimaging data (analysing [^123^I]ß-CIT-uptake) suggested that levodopa accelerates the loss of nigrostriatal DA nerve terminals or that its pharmacologic effects modify the dopamine transporter (DAT; Fahn et al. 2004). The authors interpreted this as a possibility of a levodopa-induced toxic effect. However, their calculated statistical significance for these different uptakes of [^123^I]ß-CIT was not very convincing (P = 0.036) and it should also be noted that they measured this before the treatment protocols were stopped so that a lower uptake of [^123^I]ß-CIT through DAT transporters may be explained by a competitive binding effect of higher DA concentrations in the patients receiving levodopa.

Overall, most researchers believe that, apart from the transient side effects, levodopa is safe in terms of not promoting neurodegeneration. Notably, it has been pointed out that levodopa has been shown safe in all (experimental) studies in which glial tissue was present, suggesting a protective role of glial cells (Melamed et al. 1998; Agid 1998).

### Beyond dopamine from the perspective of clinical evidence

Continuous drug delivery (CDD) is used in advanced PD to manage motor and non-motor fluctuations, particularly OFF-phase. Transdermal rotigotine, subcutaneous apomorphine infusion, and levodopa-carbidopa intestinal gel are examples of CDD aimed at providing stable dopaminergic stimulation. While effective in reducing OFF periods, these treatments do not entirely prevent them in mid-to-late-stage PD (Rota et al. 2022). Moreover, treatment with levodopa/carbidopa intestinal gel can also be associated with atypical (i.e. long-lasting) biphasic, biphasic-like (i.e. continuous), or mixed (peak-dose and continuous biphasic) dyskinesias called “complex dyskinesia” (Marano et al. 2019). The reasons for these findings are unclear, but potential factors such as drug/device-related issues, site-specific delivery problems, changes in dopaminergic and non-dopaminergic mechanisms in the basal ganglia, and more widespread network alteration including sensorimotor and cerebellar involvements have been considered (Espay et al. 2018).

Of note, continuous administration of levodopa or dopaminergic receptor agonists has been shown in MPTP-treated primates to reduce/avoid LID (Smith et al. 1997; Jenner 2002; Duty and Jenner 2011).

## Conclusion and discussion

### Conclusion

**Fig. 4:**
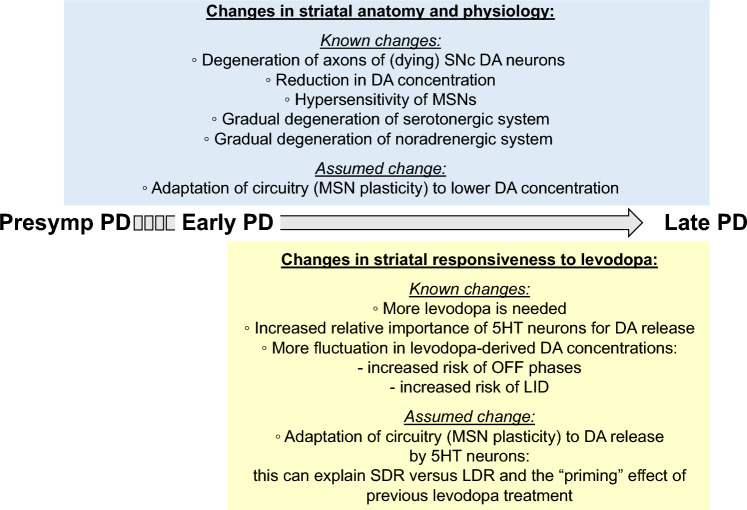
The major changes in the striatum during PD progression, including the changing responses to levodopa treatment

The use of levodopa for dopamine replacement therapy in PD has been one of the great medical discoveries of the last century. In the early phase of PD, levodopa treatment tends to work very well, frequently called the “honeymoon phase.” This probably can be explained by sufficient SNc DA neurons and axons still being present for providing endogenous DA signals and DA buffering capacity, so that levodopa treatment only has to provide “a helping hand.” It is unclear whether SNc DA neurons make a substantial quantitative contribution to the conversion in the striatum of levodopa to DA at any time during progression. However, all evidence indicates that at least during the later stages of PD, when the DA system has further degenerated, the bulk of the DA generation from levodopa in the striatum takes place in 5HT neurons that release the DA as a “false neurotransmitter” following the anatomy and signalling pattern of the serotonergic system. The 5HT neurons do not take up extracellular DA, and the absence of this buffer capacity during late PD causes—in the case of pulsatile levodopa administration in pill form—large fluctuations in striatal extracellular DA concentrations together with OFF-phase and LID challenges. While these major factors are relatively sure, it is also clear that they influence and are affected by adaptive brain plasticity, PD-induced deterioration of other neural systems, and the interference of levodopa uptake with normal 5HT release (Fig. 4). However, studies widely disagree on several of those other aspects and more research is needed.

### Clinical developments and future studies

*Subcutaneous infusion methods.* As mentioned above, continuous subcutaneous infusion with foslevodopa-foscarbidopa, a soluble formulation of phosphorylated levodopa and carbidopa, has recently been introduced (Soileau et al. 2022). Phosphorylation increases levodopa’s solubility, allowing it to be administered as an aqueous solution for continuous delivery. It has been hypothesized, however, that drugs affecting alkaline phosphatase activity, such as antidiabetics or anticonvulsants, could influence the dephosphorylation of foslevodopa/foscarbidopa to levodopa/carbidopa. This may limit the efficacy of this treatment and may contribute to a fluctuating motor response. Future real-world experience in PD patients with co-morbidities, such as diabetes, will probably provide further insights into this still hypothetical concern (Müller 2024).

Neuroderm 0612 (ND0612) is another approach to continuous subcutaneous delivery of an aqueous solution, but in this case of (non-phosphorylated) levodopa/carbidopa (LeWitt et al. 2022; Espay et al. 2024b). These subcutaneous aqueous infusion methods are easier to handle and have the potential to replace the more complex LCIG- and LECIG (LCIG plus the COMT-inhibitor entacapone) gel administration methods, which involve percutaneous endoscopic jejunostomy and are associated with a risk of severe gastrointestinal infections (Klostermann et al. 2012). The subcutaneous aqueous infusion methods probably will also become cheaper when combined with a COMT-I, i.e. opicapone (Leta et al. 2020; Müller 2024).

*Improvement of extended-release oral formulations.* Further successful developments are IPX203 as a successor of IPX066 (Hauser et al. 2011; Hauser et al. 2012; Hauser et al. 2023; Müller and Mohr 2018; Modi et al. 2019a, b; LeWitt et al. 2023; Espay et al. 2024a), reducing the number of daily levodopa/carbidopa intakes and providing a more continuous delivery of levodopa to the brain.

*Better alignment between experimental and clinical results.* Future studies in PD are likely to focus on disease-modifying approaches, such as indirect stimulation of dopaminergic neurotransmission through adenosine pathways, despite recent failures (Müller 2015; 2017; Müller and Mohr 2024). Negative translational outcomes frequently stem from poor interaction between experimental and clinical research paradigms in chronic neurodegeneration (Müller 2022). Therefore, continued improvement of PD model systems remains necessary.

*Improved monitoring of individual patients through Therapeutic Drug Monitoring (TDM).* Levodopa treatment can be optimized by monitoring plasma concentrations of levodopa in individual patients by using TDM (Hiemke et al. 2018; Müller et al. 2024). However, TDM has not yet been established in clinical practice for PD, despite its prominent use in studies aimed at optimizing the pharmacokinetic profiles of PD drugs through novel formulations and delivery methods (Modi et al. 2019a, b; Danysz et al. 2021; Marmol et al. 2021; Rosebraugh et al. 2021; LeWitt et al. 2022). In personalized medicine, TDM can reduce the risk of drug-drug interactions, optimise dose regimens, and identify issues like non-adherence or non-responsiveness (Müller et al. 2024; Titova and Chaudhuri 2017a, b). Furthermore, TDM can help differentiate between branded and generic levodopa formulations, which may vary significantly in their pharmacokinetic profiles, but sometimes are used interchangeably. For instance, in Germany, pharmacists may switch between generic and branded compounds without informing the prescribing neurologist. In summary, TDM empowers physicians to monitor—at a physiological level—the effects of variations in patient behavior, responsiveness, and drug formulations, thereby enhancing their ability to adjust treatment protocols effectively. However, whether the cost–benefit ratio of TDM in PD will justify its integration into routine clinical practice in the future remains to be determined.

### Discussion

Tremendous research has been performed since the seminal descriptions of symptoms underlying PD in 1817 by James Parkinson. Milestone papers (see Goetz 2011; Lees 2017) have been published since that time by Charcot (1892), Hassler (1938), Sano et al. (1959, 1960; Sano 2000), Carlsson et al. (1957), and Ehringer and Hornykiewicz (1960) to mention only some of them. Still, the mysteries of this devastating neurological disorder have not been solved yet, although important work has been published to get insights into the triggers of PD, into neural cell death mechanisms and into PD related genetics. The translation of Carlssons seminal research with rabbits into clinical treatment strategies of PD by using a precursor amino-acid of DA, levodopa, performed by Sano et al. (1960; see Sano 2000) in single patients with PD and by Birkmayer and Hornykiewicz (1961) as well as Barbeau et al. (1961) in open-label clinical trials has been the first solid evidence that it is possible to treat brain disorders with success. Nevertheless, there are continuous losses of neuromelanin-containing DA neurons in the SNc and of DA in the striatum. Compensatory mechanisms like enhanced DA turnover, brain plasticity and others have been suggested to overcome these neuronal disturbances and its clinical consequences for many years (Bernheimer et al. 1973).

While there is a clear beneficial response to levodopa in PD, biochemical and pharmacological studies, along with human postmortem analyses, continue to provide evidence that the underlying mechanisms of levodopa treatment are more complicated than originally assumed. This is, in part, because SNc DA neurons in the healthy dopaminergic pathway from the SN to the striatum respond to levodopa treatment through activation of various feedback mechanisms (“autoregulation”), as well as the uptake and vesicle-storage of levodopa-derived DA, ensuring a stable and continuous DA supply for striatal postsynaptic DA receptors. This buffering capacity of SNc DA neurons remains sufficiently intact during early PD, allowing levodopa treatment to largely restore motor functions.

All current evidence indicates that, in PD, levodopa is effectively synthesised and released in serotonergic presynaptic nerve terminals in the striatum. However, DA in 5HT terminals is not released under the control of an autoregulatory feedback mechanism and cannot be reabsorbed. Instead, its production and release are driven by the pulsatile increases of extracellular levodopa following medication, along with endogenous signals for 5HT release, as levodopa-derived DA is stored in 5HT vesicles. This pattern becomes more and more prominent as PD progresses and the buffering capacity of the SNc DA neurons is diminished. AIMS/LID/OFF-phases and dyskinesias are clinical symptoms associated with the pulsatile activation of striatal postsynaptic DA receptors. Studies using 6-OHDA models, aimed to elucidate the later stages of PD, have shown that the serotonergic fibre system is indeed responsible for the conversion of levodopa to DA and that it is the uncontrolled release of this neurotransmitter that causes AIMS/LID/OFF-phases and dyskinesias in experimental animals.

To avoid these side effects of levodopa treatment, Walther Birkmayer, as early as the 1960s, proposed a low-dose treatment strategy (Birkmayer and Neumayer 1972). However, in the advanced stages of PD and at higher doses of levodopa, the beneficial effects of levodopa decline and become accompanied by severe motor impairment and non-motor symptoms (Birkmayer et al. 1979; Birkmayer and Riederer 1983).

The combination of levodopa with dopaminergic D2/D3 receptor agonists, COMT inibitors, MAO-B inhibitors, AAAD inhibitors, and/or glutamatergic NMDA receptor antagonists helps to reduce the daily dose of levodopa and to avoid its potential side effects for a long period.

Even in the very advanced stage of PD, when the loss of the dopaminergic nigrostriatal pathway is (nearly) complete and the loss of the serotonergic and noradrenergic striatal innervation shows an advanced progression, levodopa has been reported to still offer some benefit. Notably, patients who have reached an end-stage of pharmacological treatment benefit often respond favourably to levodopa again after undergoing successful deep-brain stimulation (DBS), as DBS and levodopa function synergistically due to unclarified mechanisms (Vizcarra et al. 2019; Muthuraman et al. 2018).

What else to consider in these late stages of PD in regard to levodopa effects or other approaches for stimulating the dopaminergic system?

1. Not all dopaminergic systems degenerate at the same rate as the nigrostriatal system. Therefore, levodopa will be converted to DA in these systems and at higher doses may stimulate DA receptors throughout the parkinsonian brain (Brown et al. 1999; Sarre et al. 2004; Navailles et al. 2013). This may cause multiple side effects, including adverse reactions like psychotic symptoms, particularly due to the stimulation of DA receptors in mesolimbic and mesocortical brain regions.
2. The uptake of levodopa and its conversion to DA by 5HT neurons significantly impairs their 5HT production, presumably through pathway competition (see earlier). Particularly in the late stages of PD, with the 5HT system deteriorating and the levels of levodopa medication increasing, this may have a profound effect on brain function, warranting more study. The NE system may also be impacted by levodopa (see earlier), although this is less clear, as autoregulatory processes in NE neurons involving levodopa and DA probably mitigate potential effects.
  Levodopa can also impact the function of aminergic neurons by other types of competition with tryptophan, phenylalanine, or tyrosine, for example by affecting cell metabolism or during uptake across the BBB (Navailles and De Deurwaerdère 2011; Reed et al. 2012).
  The reduced/altered functionality of other neurons may be one of the reasons why LID appears in the more advanced stages of PD and not in the early phase (Cenci and Konradi 2010; Fieblinger et al. 2014; Shen et al. 2016; Sciacca et al. 2023), while NMS are early, even prodromal signs of PD (Birkmayer and Riederer 1983).

3) Levodopa may also be converted to DA in non-neural cells possessing AAAD, such as glial and endothelial cells. This DA could stimulate DA receptors throughout the brain, including in the striatum. In this context, MAO-B inhibitors may be beneficial, as they are expected to help increase DA production not only by aminergic systems but also by other potential DA-producing systems (Konradi et al. 1988; Riederer et al. 1989).
4) Inhibition of the dopamine transporter (DAT) molecules represents another possible approach to enhance striatal as well as extra-striatal DA concentrations (Abercrombie et al. 1990; Zigmond et al. 1990). Indeed, DA-uptake inhibitors have been shown to help improve both motor and non-motor symptoms of PD (Nutt et al. 2004).
5) Selective D2/D3 receptor agonists might still be successful in stimulating postsynaptic striatal receptors in the case of a total loss of striatal presynaptic dopaminergic terminals (Maggio and Millan 2010; Isaacson et al. 2023). However, such receptors are not only restricted to the dorsal striatum, and non-motor side effects, like psychoses, can be the consequence. Therefore, and because of the long half-life time of DA-receptor agonists and the better tolerability of levodopa, levodopa is the preferred treatment in late PD and after deep-brain stimulation.
6) Glutamatergic receptor antagonists may have balancing effects on the cortico-striatal input as well as on the output of the motor loop (Kornhuber et al. 1991; 1995; Lange et al. 1997; Johnson et al. 2009).

Finally, we would like to address several critical limitations that impact our current understanding of PD pathology and the impact of levodopa.

Firstly, there is a scarcity of postmortem data conclusively characterizing the PD-stage-dependent deterioration pattern for each of the aminergic systems. This is partly due to strict governmental regulations for autopsy studies (Conolly et al. 2015) and the heterogeneity among PD subtypes, each with distinct pathological features (Birkmayer et al. 1979; Höglinger et al. 2024; Strobel et al. 2024). Additionally, the anatomical routes of initial PD progression vary between patients, encompassing both gut-brain and retrograde degeneration pathways (Borghammer and Van Den Berge 2019; Wüllner et al. 2023 for review).

Secondly, only some information about a phase-dependent progression of the pathology of PD can be drawn from either human imaging studies or animal models of PD. Human imaging studies have been primarily focused on assessing nigrostriatal dopaminergic deficits at the onset of symptoms and in advanced stages of PD (see Table 2). However, further studies are needed to investigate the phase-dependent pathology of serotonergic, noradrenergic and glutamatergic systems innervating the striatum.

Thirdly, despite numerous experimental animal studies using toxin-induced damage of the SN and/or the striatum with a variety of experimental designs (see for reviews Boulet et al. 2008; Lindgren et al. 2010; Duty and Jenner 2011; Navailles et al. 2013; and Cenci 2014; to mention a few ones), the investigation of human PD-mimicking phase-related early vs. late pathology combined with respective analytical methodology has been a near impossible exercise. Therefore, these experimental studies all lack one or more important data, such as those that can be obtained by (multisite) intracerebral microdialysis, (immune-) histochemistry, levodopa-treatment, multiple transmitter system lesions, consideration of laterality, and/or early PD phase designs. As a result, it is difficult to draw definite conclusions about how levodopa treatment and disease progression interact.

Fourthly, an unsolved question remains. How do levodopa or striatal DA generated from levodopa via 5HT neuronal support affect the feedback mechanisms in surviving SNc DA neurons? Do they largely inhibit endogenous DA synthesis in these neurons, and if so, is such effect toxic, neutral, or even neuroprotective?

In summary, the available data from clinical studies, human imaging studies, postmortem neuropathology and numerous experimental studies using various methodological designs collectively support that low-dose levodopa treatment in the early phase of PD pathology is generally safe and without severe side effects. Only at more advanced stages of the disease does the risk of LID increase due to the progressive loss of dopaminergic striatal innervation. At this point, DA production relevant for the striatum predominantly arises from levodopa converted to DA within serotonergic striatal terminals, with lesser contributions from noradrenergic striatal terminals and potentially also from glial and endothelial cells. Ideally, these different unnatural sources of levodopa-induced dopaminergic action should sufficiently mimic DA release in the healthy striatum to help prevent the abnormal involuntary movements and non-motor symptoms typical of PD. It is only logical that this mimicking threshold cannot be achieved perfectly in the late disease stages when the support from surviving SNc DA neurons further dwindles. Nevertheless, the extent to which levodopa treatment can prevent many PD symptoms for a long period of the disease remains amazing.

Ultimately, we must move beyond the simplistic view of the brain as a machine and levodopa as a fuel. The brain’s systems are far more complex, flexible and adaptable.

## Abbreviations

5HT: 5-Hyroxytryptamine/serotonin/serotonergic
5HTP: 5-Hydroxtryptophan
6-OHDA: 6-Hydroxydopamine
AADC: Aromatic L-amino acid decarboxylase
AADC-I: Aromatic L-amino acid decarboxylase inhibitor
BBB: Blood brain barrier
CA: Catecholamine, catecholaminergic
COMT: Catechol O-methyl transferase
COMT-I: Catechol O-methyltransferase inhibitor
DA: Dopamine/dopaminergic
DAT: Dopamine transporter
L-DOPA: L-3,4-dihydroxyphenylalanine
GCH1: GTP cyclohydrohyrolase I
LC: Locus Coeruleus
LDR: Long duration response
LID: L-DOPA-Induced dyskinesia
LTD: Long-term depression
LTP: Long term potentiation
MAO-B: Monoamine oxidase B
MAO-B-I: Monoamine oxid B-inhibitor
MSN: Medial spiny neuron
NE: Norepinephrine/noradrenaline/noradrenergic
PD: Parkinson’s disease
PET: Positron emission tomography
STD: Short term response
SN: Substantia nigra
SNc: Substantia nigra pars compacta
SNr: Substantia nigra pars reticulata
SPECT: Single photon emission tomography
TH: Tyrosine hydroxylase
VTA: Ventral tegmental area
WT: Wild type
